# Structurally and Functionally Adaptive Biomimetic Periosteum: Materials, Fabrication, and Construction Strategies

**DOI:** 10.1002/EXP.70005

**Published:** 2025-02-27

**Authors:** Yuhan Du, Yujie Liu, Yuanchi Zhang, Yangyi Nie, Zili Xu, Ling Qin, Wei Zhang, Yuxiao Lai

**Affiliations:** ^1^ Centre for Translational Medicine Research and Development Shenzhen Institute of Advanced Technology Chinese Academy of Sciences Shenzhen China; ^2^ University of Chinese Academy of Sciences Beijing China; ^3^ School of Biomedical Sciences and Engineering South China University of Technology Guangzhou International Campus Guangzhou China; ^4^ National Innovation Center for Advanced Medical Devices Shenzhen China; ^5^ Department of Orthopaedics and Traumatology The Chinese University of Hong Kong Hong Kong China; ^6^ Guangdong Engineering Laboratory of Biomaterials Additive Manufacturing Shenzhen China

**Keywords:** biomimetic periosteum, biomimicry, bone defect repair, materials, techniques

## Abstract

The periosteum is crucial in the processes of bone formation, regeneration, and remodelling. Specifically, periosteal progenitor cells contribute a major force to the initiation of bone healing. Biomimetic periosteum (BP), employed for treating bone defects, exhibits superior outcomes in terms of bone integrity, proper vascularization, and minimal heterotopic ossification when compared to conventional direct graft bone void fillers. Therefore, BP has emerged as a contemporary and effective approach for addressing bone defects. As an in vivo graft, BP necessitates excellent biocompatibility and appropriate mechanical properties. Furthermore, it should closely mirror the architecture and functionality of the natural periosteum. This review provides a detailed summary of recent research progress on BP, incorporating inspiring studies that contribute to the future development of this field. Initially, the review examines the structure and function of the periosteum in the context of bone defect repair. Subsequently, it analyzes the current research and design concept for BP construction and provides a comprehensive overview of the materials and techniques employed in constructing BP. Finally, it summarizes the construction strategies of BP used for treating bone defects from various perspectives including structural and functional biomimicry, and discusses the latest advances in current research.

## Graphical abstract

Schematic illustration of structural and functional biomimicry. The upper part shows BP with various structures such as monolayered, surface‐microstructural, bilayered and multilayered. The bottom part exhibits BP with different functions including immune regulation, drugs/cells/factors/ions loading, as well as bioelectrical stimulation and neuroregulation.

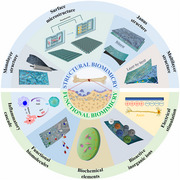

## 1 Introduction

The periosteum, a vital connective tissue enveloping bones, is indispensable for bone development and repair. It consists of an outer fibrous layer and an inner cambium layer, which together create a nurturing microenvironment for bone growth and healing. The periosteum's osteogenic progenitor cells play a crucial role in the healing of bone defects, rapidly differentiating to drive bone repair [1, 2, 3]. Although natural periosteum transplantation is clinically used to assist bone healing, its limitations, such as restricted availability, high morbidity at the donor site, and potential immune rejection, significantly hinder treatment efficacy [4, 5]. In response to this challenge, biomimetic periosteum (BP) has emerged as a promising technology to overcome these limitations. It is worth mentioning that bone healing induced with BP also exhibited superior bone integrity, proper vascularization, and minimal heterotopic ossification compared to bone void fillers [6, 7].

In recent years, continuous progress has been made in biologically inspired research for constructing BP. Numerous BP designs have successfully replicated the hierarchical macroscopic and microscopic structures of natural periosteum through careful selection of materials and various preparation techniques [8]. An example of BP that has found clinical use in the context of guided bone regeneration is the Geistlich Bio‐Gide collagen membrane. Although primarily utilized in dental applications, the principles of tissue regeneration and new bone formation demonstrated by Geistlich Bio‐Gide are relevant to the broader concept of BP in orthopedics. Consequently, the clinical application of BP, including collagen membranes like Geistlich Bio‐Gide, has become an area of active research in orthopedic surgery, with studies exploring their potential for enhancing bone repair and regeneration [9, 10].

However, the high degradation rate and weak mechanical properties of Bio‐Gide membranes lead to membrane perforation during implantation, which greatly affects the repair effect [11, 12]. As the field of BP continues to evolve, there is a growing recognition of the need to develop more advanced and multifunctional BP constructs to address the limitations of current materials and techniques. This includes exploring new biomaterials, adopting advanced manufacturing methods (such as 3D printing and electrospinning), and integrating components with high mechanical performance, degradation cycles that can match the bone repair cycle, and promote angiogenesis, osteogenesis, and innervation [13, 14]. The ultimate goal is to create BP constructs that closely approximate the complex structure and function of the natural periosteum, providing a more effective solution for bone repair and regeneration [15, 16].

**SCHEME 1:**
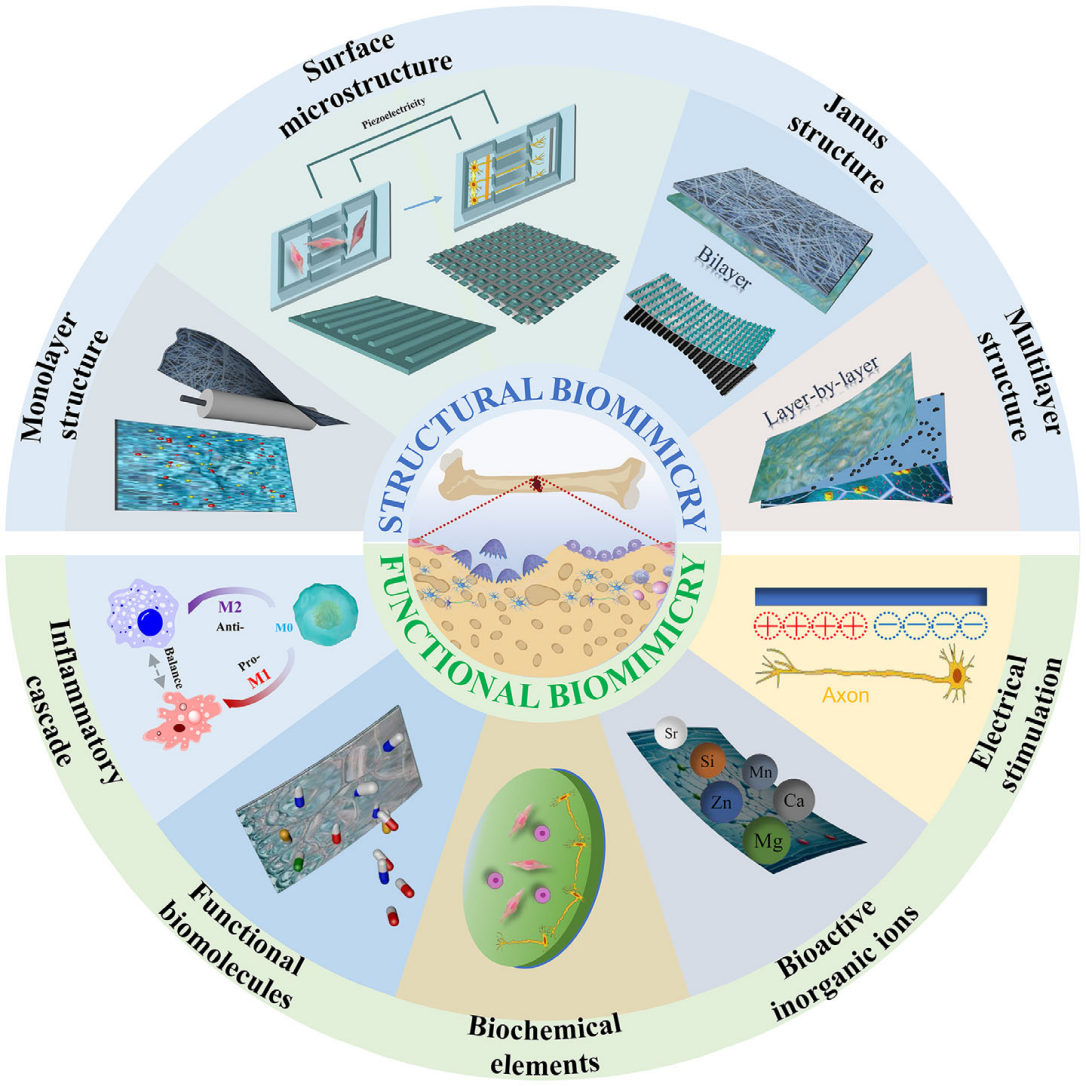
Schematic illustration of structural and functional biomimicry. The upper part shows BP with various structures such as monolayered, surface‐microstructural, bilayered and multilayered. The bottom part exhibits BP with different functions including immune regulation, drug therapy, cells/factors/ions loading, as well as bioelectrical stimulation and neuroregulation.

In this comprehensive review, we connect to the emerging bionic database that comprehensively summarized the structural and functional BP‐assisted bone defect repair, the significant advances of which have not been reported yet to the best of our knowledge (Scheme 1). We commence by examining the structure and function of periosteum. Subsequently, we delve into the principal strategies employed in constructing BPs, encompassing the materials and techniques involved in their construction, as well as the structural and functional bionics of BPs. Lastly, we highlight the previously overlooked role of intraperiosteal neural tissue in the bone repair process. Additionally, we explore potential strategies aimed at stimulating both bone and nerve repair to enhance long‐term outcomes, drawing insights from existing literature.

## 2 Structure and Function of the Periosteum

**FIGURE 1:**
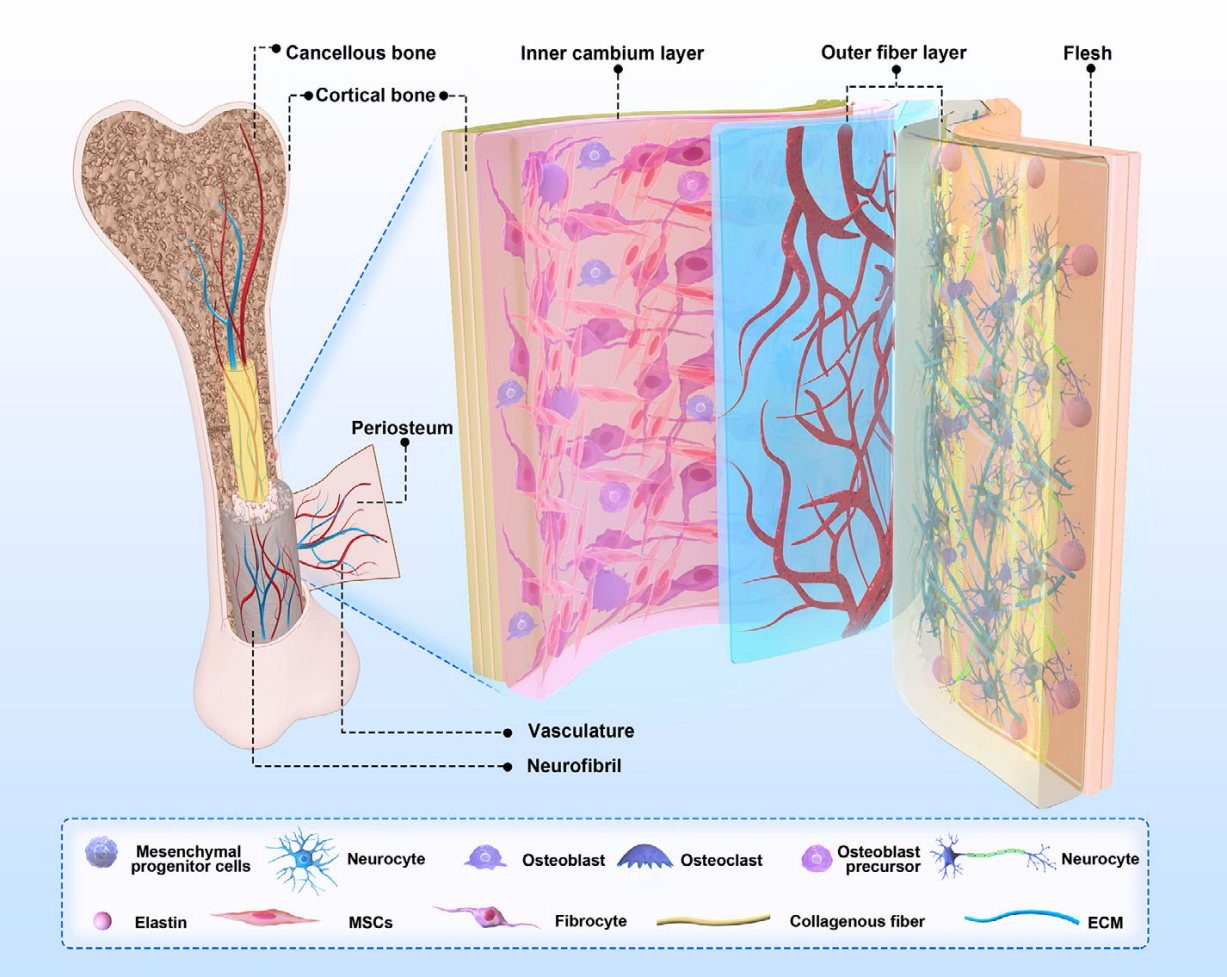
Schematic structure of the natural periosteum.

The periosteum constitutes a pivotal layer of connective tissue that enwraps the entire skeletal surface, excluding the joint regions. Its significance lies in its essential role in osteogenesis and the repair of bone defects [17]. Comprising dual strata, the exomorphic fibrous lamina and the endomorphic generative cambium, this connective tissue gives rise to a sophisticated bio‐interface that bestows skeletal fortification, preservation, and reparative functionalities [18]. In Figure 1, the layered structure of the periosteum is detailed.

The outer fiber layer indeed predominantly comprises densely interwoven collagen fibers, elastin, and extracellular matrix (ECM) that intricately intertwine with the bony matrix [19]. These collagen entities confer robustness and constancy upon the periosteum, thus imbuing the skeletal framework with structural resilience. Notably, the peripheral periosteum exhibits profuse vascularity, manifested by an intricate vascular plexus that extends from its matrix into the medullary recesses via conduits that transgress the osseous contour [20]. This intricate vascular meshwork assures the efficient distribution of oxygen, nutrients, and cellular entities to osseous tissues, thus expediting their metabolic kinetics and reparative modalities [21]. By virtue of its pivotal role in vascular provisioning, the periosteum is frequently likened to the “umbilical cord” of the cortical osseous continuum. The inner cambium, residing beneath the exomorphic fibrous periosteum, constitutes an assemblage of diverse cellular cohorts. These encompass mesenchymal progenitors, differentiated osteogenic precursors, osteoblasts, fibroblasts, and undifferentiated periosteal stem cells [22]. Their concerted efforts are dedicated to overseeing osteogenesis and orchestrating osseous regeneration. Importantly, it is observed that the sensory and autonomic nerves, positioned between the periosteum's outer fibrous layer and the inner layer enriched with stem cells, play essential roles in bone metabolism regulation. More significantly, in comparison to mineralized bone or bone marrow, the neural network within the periosteum manifests a more compact configuration, assuming a pivotal role in perceiving and regulating pain as well as tactile signals. It is widely held that the periosteum's neural fibers are primarily unmyelinated, with their bare nerve endings being closely involved in the detection of pain [23, 24]. Certain nerve fibers traverse the bone shaft via Volkmann's canals, while the remainder establish nerve endings within the periosteum. Immunocytochemical investigations have unveiled the presence of substance P‐stained neural plexuses within the periosteum [25]. In immediate proximity beneath the periosteal surface, minuscule nerve fiber branches containing substance P may be correlated with the pain sensitivity of the periosteum. The elaborate network of nerve endings encircling the periosteum excels in discerning and transmitting pain stimuli, instigating a cascade of defensive responses designed to shield the affected region from potential harm, and concurrently implementing cautious intervention measures. Consequently, this succession of responses prompted by pain constitutes the fundamental mechanism of individual protection, accentuating the imperative requirement for timely intervention [26, 27].

**FIGURE 2:**
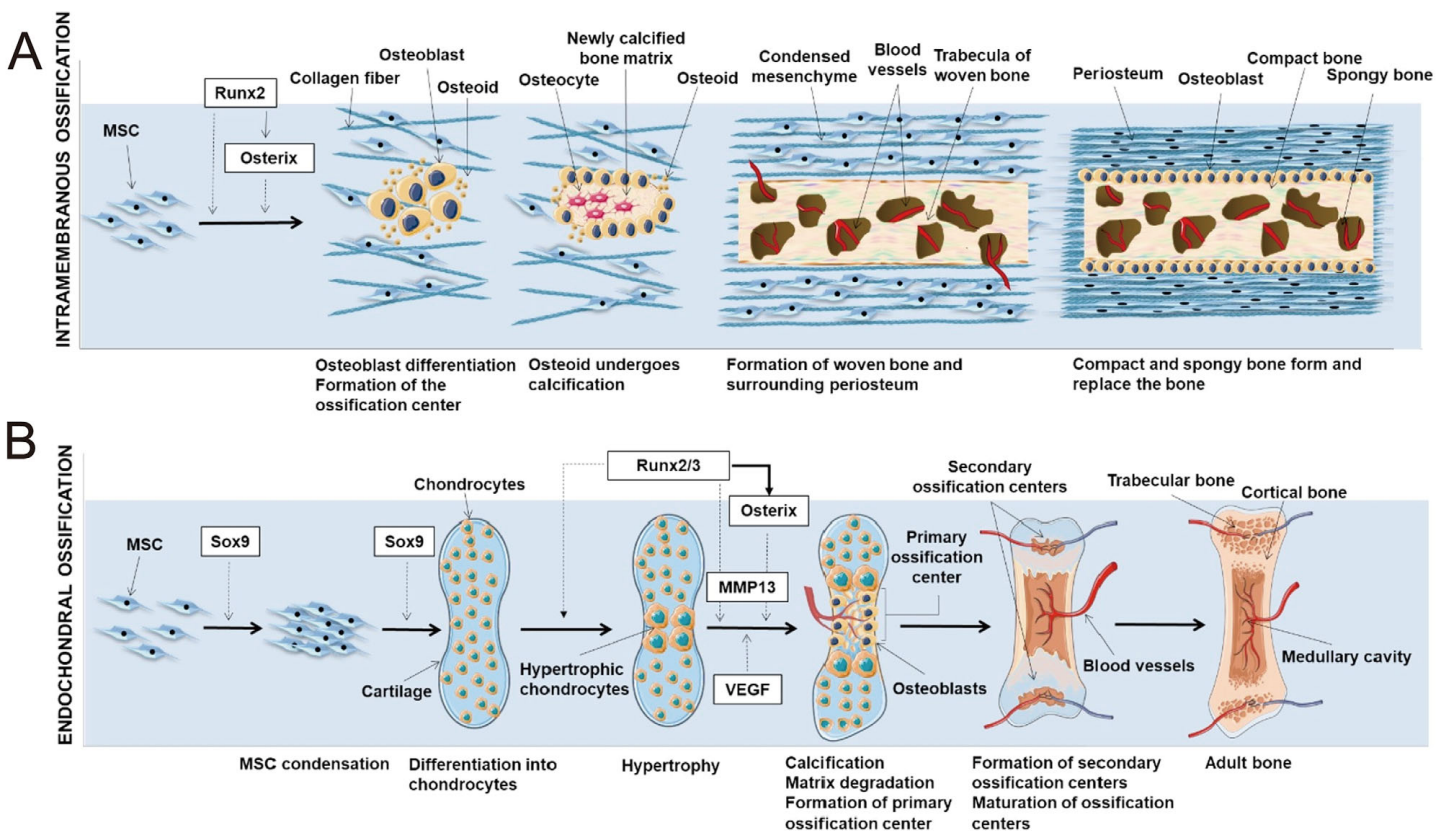
Two ways of mediated osteogenesis. (A) In the depicted process of intramembranous ossification, the preliminary step involves MSCs clustering and transitioning into osteoblasts, which establish ossification centers. Runx2 is critical for osteogenic differentiation, influencing it directly or through the delayed triggering of Osterix expression. Subsequently, osteoblasts start synthesizing a bone‐analogous substrate that rapidly calcifies. Osteoblasts within this calcified structure mature into osteocytes. The periosteum emerges as vascularized mesenchyme aggregates at the woven bone's periphery, leading to woven bone formation with vascularized internal areas that will transform into a marrow cavity. The trabecular surfaces accumulate matrix, consolidating into dense bone, while spongy bone remains within. (B) Schematic representation of endochondral ossification. For endochondral ossification, MSCs post‐coalescence specialize into chondrocytes, laying down a cartilage framework. Central chondrocytes swell, indicating hypertrophy, a process regulated by Sox9 and the Runx2/3 complex, essential transcription factors for chondrocyte development and expansion. These hypertrophic chondrocytes invite vascular growth. Osterix serves a dual role, facilitating Runx2/3's functions during the calcification of cartilage and matrix decomposition and prompting MMP13 production. Osteoblasts evolve from cells that infiltrate the cartilage mold into areas of vascular incursion, starting bone creation at primary ossification zones. Bone growth propagates along the longitudinal axis, establishing secondary ossification sites. Ultimately, this culminates in the formation of mature bone, characterized by trabecular and cortical structures, along with a central medullary space [30]. Copyright 2018, Elsevier.

The biological processes orchestrated by the periosteum during embryonic bone development and postnatal bone growth are of paramount importance. Undifferentiated progenitor cells undergo intramembranous ossification to form periosteal tissue during embryonic bone development, while the perichondrium in the surrounding area primarily follows endochondral ossification pathways [28]. Subsequent to these events, regulatory mechanisms come into play, guiding the transformation of perichondrium into periosteum, thereby promoting further growth of the skeletal structure [29]. Postnatally, bone tissue continues to undergo growth, and the periosteum functions as a reservoir of cells, fulfilling a vital function in the progression of bone formation. Throughout the entire life cycle, the periosteum not only facilitates the normal growth and development of bones but also actively participates in bone restructuring and healing as a response to external stimuli. In instances of bone injury leading to intramembranous ossification, mesenchymal stem cells (MSCs) will transform into osteoblasts found within the mesenchymal or medullary spaces (Figure 2A). This process is mainly accountable for the development of the flat bones of the skull and the partial formation of the clavicle during early childhood. On the other hand, endochondral ossification (Figure 2B) is different in that the chondrocytes derived from the surrounding cartilage tissue are initially responsible for forming the matrix template, originating from the growth plate, subsequently transforming into bone structures. This ossification process is instrumental in the embryonic development of the body's long bones [30]. The onset of intramembranous ossification is marked by the emergence of a bone defect—a cluster of undifferentiated MSCs. These cell aggregates cease proliferation, transition into osteoblastic phenotypes, and ultimately mature into osteoblasts via a transitional pre‐osteoblastic stage. However, as age advances, the periosteum undergoes gradual degeneration, leading to a diminishing osteogenic potential [31].

The cellular constituents of the periosteum are collectively referred to as periosteum‐derived cells, which include periosteum‐derived stem cells (PDSCs) [32]. These cells demonstrate excellent capabilities in terms of proliferation, osteogenic differentiation, and angiogenesis. Researchers have effectively isolated periosteum‐derived cells, such as CD90+ cells, and have observed their superior proliferative and osteogenic potentials in both in vivo and in vitro settings [33]. Additionally, the perivascular cells within the periosteum not only induce vascular formation to aid in bone regeneration but also undergo differentiation into osteoblasts during the activation process, contributing to the osteogenic cell lineage. Furthermore, the ECM of the periosteum is of substantial importance in the osteogenic process [34]. It provides the necessary microstructures and biochemical cues, facilitating cellular adhesion, proliferation, and differentiation. Moreover, it collaborates in facilitating decellularized mineralization during bone formation, promoting in situ regeneration of bone defects and ectopic ossification. Numerous growth factors, including vascular endothelial growth factor (VEGF) and bone morphogenetic protein 2 (BMP‐2), are critical in the processes of new blood vessel formation and bone development [35]. In conclusion, the ECM of periosteal cells, along with its alternatives, holds great potential for tissue engineering applications and innovations. The highly vascular nature of the periosteum is crucial for maintaining the structural integrity and functionality of bone tissue. An adequate blood supply not only fulfills the metabolic demands of the periosteum but also but also extends nourishment to the bone tissues nearby via its vascular network [36, 37]. A stable blood supply is imperative for periosteal bone formation post fractures. The rapid establishment of new blood vessels significantly contributes to the formation of new bone, playing a extremely important role in the overall healing of bone tissue. The nervous system plays a crucial role in governing and coordinating the activities of the immune, vascular, and skeletal systems within the bone microenvironment [38, 39]. A study conducted by Qin's team revealed that the blockade of sensory nerves in the periosteum resulted in a deceleration of bone healing [40]. This phenomenon is primarily attributed to the direct impact of sensory nerves on hematopoietic stem cells (HSCs) through the secretion of neuropeptides such as calcitonin gene‐related peptide (CGRP), receptor activity modifier protein 1 (RAMP1), and calcitonin receptor‐like receptors. The activation of the Gαs/adenylate cyclase/cAMP pathway further stimulates downstream HSCs [41]. Additionally, various neuropeptides, including CGRP, SP, and vasoactive intestinal peptide (VIP), released by sensory nerves, can enhance bone remodeling by stimulating diverse signaling pathways such as Wnt and Sonic hedgehog [42].

## 3 Design and Construction of Ideal BP

The choice of material serves as the foundation for constructing BP, demanding excellent compatibility with adjacent tissues and cells to establish a surface conducive to cell adhesion, proliferation, and differentiation. In the endeavor to attain an ideal BP, it is crucial to deliberate upon surface micro‐ and macro‐structures that closely mirror the intrinsic architecture of the native periosteum [43]. This replication is imperative to emulate the physiological milieu of bone cells and augment their typical functionality. Accordingly, optimizing the surface structure of BPs, including the construction of monolayers, multilayers, anisotropic micro/nanostructures, and composite BPs, holds significance in fostering bone regeneration and osseointegration. Prior investigations have demonstrated that the surface microstructure of BPs influences specific cellular responses, including adhesion, proliferation, and osteogenic differentiation [44]. Moreover, serving as an intermediary interface between bone and muscle, the periosteum necessitates the essential attributes of strength and flexibility to endure the biomechanical forces it encounters and sustain its structural integrity over an extended duration [45]. Adequate mechanical properties can facilitate the establishment of an optimal microenvironment for bone regeneration. This process not only stimulates the development of local blood vessels but also bolsters the mobilization of osteoblasts and the creation of a mineralized cartilage matrix [46]. Matsumoto and colleagues have shown that the mechanical attributes of BP are integral to providing efficient biophysical signals for improved bone healing. An increase in mechanical stress has been noted to enhance the regenerative density at sites of bone defects. In parallel, it boosts the synthesis of prostaglandin (PG)E2, a vital compound that influences the growth and structural modification of bone cells [47]. Simultaneously, to restrain the growth of adjacent scar tissue, it is imperative to employ a BP characterized by robust adhesive properties, ensuring secure adherence to the defect site under both dry and wet conditions.

Moreover, the internal immune milieu and periosteal topology undergo changes throughout the dynamic bone repair process. Following bone injury, the periosteum undergoes thickening due to the periosteal reaction, particularly in the periosteal layer [48, 49]. Subsequently, as bone progenitor cells proliferate, the periosteum assumes a distinct fascicular structure. The resulting periosteal callus develops a wavy topology, evolving into woven bone and eventually forming a complete lamellar bone. This formation imparts the periosteum with a nuanced surface structure and vertically oriented extracellular matrix (ECM) fibers that can influence cellular alignment, collagen fiber formation, and osteogenesis [50]. Essentially, the simulated reconstruction of periosteal morphology holds significant promise. The design of functional BP often draws inspiration from the dynamics of the periosteal microenvironment during bone repair and the tissue microenvironment post‐fracture or bone defect. This intricate process involves the generation of immune cells in the inflammatory cascade, various biochemical factors, inorganic ions (e.g., calcium, phosphorus, magnesium, and manganese), regenerative cells, osteogenic and angiogenic factors, and bioactive molecules such as nanomedicines. Crucially, BP incorporates innervation, angiogenesis, and osteogenesis. Consequently, BPs should be designed considering these factors to effectively enhance fracture/defect healing. Optimizing BP components, utilizing materials that promote the secretion of osteoblasts and angiogenic cells or related growth factors, and loading them with functional components (e.g., analogous cells or growth factors, inorganic ions, or therapeutic drugs) are crucial steps in enhancing the functionality of BPs for bone regeneration and osseointegration.

## 4 Materials and Techniques for Construction BP

Currently, a variety of materials, including tissue‐ or cell‐derived substances, natural polymeric materials and synthetic polymers, have been used to manufacture BPs for the treatment of bone defects or fractures [51, 52, 53]. This chapter examines the current sources of materials and techniques for BP in three primary categories, as indicated in Table 1.

**TABLE 1:** Various materials and technology for constructing BP.

Classification	Materials and technology	Experimental model	Characteristics	Ref.
**Decellularized extracellular matrix and cell sheets**				
Tissue‐derived dECM	Decellularized periosteum: Decellularization technology	In vitro cell validation	Hydrogels derived from dECM retain the biological composition and function of natural ECMs and can provide tissue‐specific clues to regulate phenotypic expression and cell fate	[57]
	Intestinal submucosa/MAGel/HAp: Biomineralization and chemical crosslinking	In vitro cell validation	The expression of M2 marker is up‐regulated, and the osteogenic differentiation of PDLSC is enhanced by BMP‐2/Smad signaling pathway	[58]
	Human amniotic membrane	Tibial diaphyseal defects	Amniotic membrane contains a variety of physiologically active substances that promote angiogenesis and stem cell differentiation	[60]
	Acellular human dermis	Segmental bone defect	It Allows cell regeneration, vascular reconstruction and bone defect repair to transport cells or bone‐induced proteins for periosteal regeneration	[118]
Cell‐derived dECM	MSCs dECM: Decellularization method	In vitro cell validation	Cell‐derived dECM does not stimulate the host immune response and has no limitations such as pathogen transfer	[63]
	Fibroblast dECM: Decellularization method	In vitro cell validation	The adipose‐derived MSCs were induced to differentiate into cartilage	[64]
	Preosteoblast dECM: Decellularization method	In vitro cell validation	The BP promotes cell adhesion and proliferation, thereby stimulating osteogenic differentiation of MSCs	[65]
	Cartilage cell dECM: Decellularization method	Canine articular cartilage defect	These membranes are highly compatible with cells and do not show any cytotoxicity or immune response in vivo	[119]
Cell sheets	Osteoblasts sheet	Calvarial defect	The BP promotes bone formation in vivo through bionic natural periosteum	[66]
	MSCs sheet	Femur defect	MSCs sheet induce prolonged cartilage formation and enhanced bone callus formation at the graft‐host junction, as well as graft‐host bone integration	[67]
	BMSCs sheet + HUVECs sheet	In vitro cell validation	The promotion of vascular network generation by BP in vitro was functionally consistent with the angiogenesis in vivo in mice	[68]
	Osteoblasts sheet and endothelial‐like cells sheet	Radius bone defect	The BP is similar to the natural periosteal composition and can simulate the periosteal environment to promote bone formation and angiogenesis in vivo	[120]
	Preosteoblast sheet + BMSCs	Posterior spinal cord lesions	This BP enhances bone repair through a bionic periosteal microenvironment	[95]
**Natural polymer**				
Collagen	Collagen + Chitosan/heparin/bFGF: Hydrogels	In vitro cell validation	This double‐layer collagen membrane has the spatial and temporal control of growth factor delivery, mild manufacturing conditions and simple process, and has potential application prospects in the field of tissue repair	[74]
	Collagen + Carboxymethyl chitosan: Hydrogels	Calvarial defect	Collagen fibers can not only simulate the structure of periosteal fiber layer as a shielding membrane, but also coordinate the release of Il‐4 to promote the polarization of M2 macrophages to reconstruct the local microenvironment, thus promoting angiogenesis and osteogenesis	[75]
Gelatin	Gelatin + Oxidized HA + Micro/nano bioactive glass: Organic/inorganic co‐crosslinked hydrogels	Calvarial defect	Organic–inorganic BP accelerates the vascularization of the defect area and collaboratively promotes the repair of bone defect	[76]
	Gelatin‐diselenide + Calcium alginate: Organic/inorganic co‐crosslinked hydrogels	Calvarial defect	This BP enhances periosteum stability, anti‐swelling and delayed degradation. It can continuously release nitric oxide (NO), activate nitric oxide circulating guanylate (NO‐CGMP) signaling pathway, coordinate the coupling effect of angiogenesis and osteogenesis, and accelerate bone repair defects	[45]
GelMA	GelMA + Porous bioactive glass: Organic–inorganic double crosslinking technology	Calvarial defect	The gel membrane formed by organic‐inorganic crosslinking not only simulates the periosteal microenvironment to promote angiogenesis and osteogenesis, but also enhances the mechanical strength and further plays a role in the subsequent bone reconstruction	[79]
	GelMA + CaPs + HUVEC + MC3T3‐E1: Electrospinning + Hydrogels	In vitro cell validation	Its mimicking the periosteal environment has the potential to promote angiogenesis and osteogenesis	[80]
Chitosan	Chitosan multilayers/foam/nanofibers: Electrospun/freeze dried/polyelectrolyte multilayers	In vitro cell validation	The periosteum‐mimicking scaffolds support bone progenitor cell phenotypes, which can be used as periosteal mimics to deliver bone progenitor cells and improve the healing of allogeneic bone	[121]
	Chitosan + Alginate + BMP‐2: Layer‐by‐layer assembly	In vitro cell validation	The BP mimics a regulated periosteal microenvironment and stimulates stem cell proliferation, migration and differentiation through BMP	[122]
	Chitosan + Xanthan: Chemically modified hydrogels	In vitro cell validation	The BP will be able to concentrate natural BMP and induce bone formation	[86]
Alginate	Alginate + HAp: Organic–inorganic double crosslinking technology	Subcutaneously on the back	The BP has a highly porous fiber layer and a mineral‐rich active layer, and has the activity of promoting bone cell proliferation and differentiation and promoting fibroblast‐like cell proliferation	[123]
	Alginate + CaCl_2_: Crosslinked hydrogels	In vitro cell validation	The gel system supports periosteum‐derived cartilage formation, and its injectable delivery can be used to fill complex defects in joint surfaces through minimally invasive procedures	[124]
**Synthetic materials**				
PLGA	PLGA/MgO/Quercetin: Electrospinning technology	Calvarial defect	PLGA/MgO membranes with different quercetin concentrations have a variety of biological activities, Mg^2+^ can promote the secretion of CGRP and affect the neuroregulation of the bone regeneration process	[99]
	PLGA: Capillary force lithography in combination with a surface micro‐wrinkling method	In vitro cell validation	The BP is attached to different scaffolds and implants with a high degree of stability and adhesion in the water environment and promotes the expression of osteogenic markers of cultured stem cells	[125]
PLLA	Hyaluronan‐PLLA + VEGF: Collagen self‐assembly + Micro‐sol electrospinning	Calvarial defect	This BP has a layered structure that not only mimics the bone repair microenvironment but also promotes angiogenesis through the sustained release of VEGF	[93]
	HA + PLLA + bFGF+COL‐1: Collagen self‐assembly + Micro‐sol electrospinning	In vitro cell validation	Micro/nanofiber scaffolds induce endogenous regeneration mechanisms by mimicking the microstructure of natural atrial fibrillation tissue	[126]
PCL	PCL + Collagen + HAp: Electrospinning technology	Femur segmental defect	The BP restored bone formation in the periosteum at the donor site, thereby reversing the poor biomechanics of allograft bone healing 6 weeks after implantation	[89]
	PCL + Gelatin + Icariin: Electrospinning technology	In vitro cell validation	BP was produced by electrospinning to enhance hydrophilicity, mechanical strength, degradation rate, and biocompatibility, and the loading of ICA also enhanced bone regeneration	[127]
PU	PU + PCA + Zn: One‐pot deposition method	In vitro cell validation	A novel BP with excellent biocompatibility and regulation of oxidative stress conditions, promoting bone formation and mineralization to promote bone regeneration	[94]
	PCL + co‐PUPCL + HAp: Conjugate electrospinning technology	In vitro cell validation	Tests of cell infiltration on both sides of the three membranes in vitro and in vivo showed that the inner layer of BP had good cell permeability deep inside the scaffold, while the outer layer blocked the cells	[95]
PEG	PEG hydrogels + MSCs: Hydrogels	Femur defect	This BP localization of MSCs to the surface of acellular bone allograft can directly promote osteogenic differentiation of stem cells	[128]
	PEG hydrogels + MSCs + Osteoprogenitor cells: Hydrogels	Femur defect	This BP mimics the natural periosteal cell population and produces sufficient paracrine factors to synergically promote bone regeneration	[129]
PACG	PACG‐GelMA‐Mg^2+^+GelMA‐HAp: 3D printing	Calvarial defect	GelMA‐HAp layer has the function of promoting osteogenic differentiation of rat BMSCs while sustainably releasing Ca^2+^. The PACG‐GelMA‐Mg^2+^ layer can protect the internal defect site and prolong the degradation time and released magnesium ions to regulate the polarization of macrophages into M2 phenotype and promote the angiogenesis of HUVECs in vitro	[130]
PVDF	TiO_2_@PVDF nanofiber membranes: Electrospinning technology	In vitro cell validation	The osteoblastic differentiation of piezoelectric fiber membrane BP was much higher than that of simple BP	[52]
SMP	Poly(ε‐caprolactone)‐diacrylates (PCLDA) with different molecular weights: 4D printing	Femur defect	The SMP layer has a responsive surface microstructure that can precisely switch the stages of proliferation and differentiation, thus promoting bone formation. The hydrogel layer gives the membrane the ability to digitally adjust its 3D geometry to match specific macroscopic bone shapes in a clinical scene	[131]

### 4.1 Decellularized Extracellular Matrix and Cell Sheets

Decellularized extracellular matrix (dECM), also known as decellularized matrix material, stands as a pivotal material in the fields of bone tissue engineering. This distinctive material is subjected to a thorough decellularization procedure that guarantees cellular removal, yet retains the essential bioactive elements, which are fundamental to the complex makeup and architecture of the extracellular matrix. Serving as a highly coordinated organic entity, dECM houses various signaling molecules within its matrix. Encompassing essential ECM components such as collagen, elastin, mucopolysaccharides, active peptides, and growth factors, this matrix is distinguished by its remarkable attributes of high biocompatibility, bioactivity, and degradability [54]. Its multifaceted nature empowers dECM to induce and promote critical cellular processes, including adhesion, proliferation, differentiation, and tissue formation. Consequently, dECM became the cornerstone for the construction of putative osteochondral materials.

Tissue‐derived dECM are primarily sourced from animals or humans, including periosteum, intestinal mucosa, amniotic membrane, and dermal matrix. These natural membranes can undergo decellularization through physicochemical methods and enzymatic processes to shape biomimetic platforms matching bone defect areas [55]. Significantly, dECM possesses a rich and naturally ordered collagen fiber network, making it a promising material for constructing periosteum substitutes. Lin et al. discovered that the dECM periosteum can serve as a template to influence the behavior of crystals during biomineralization, thereby controlling the size and shape of growing apatite particles. Moreover, this dECM has the capability to regenerate through bone defects [56]. Li et al. created a decellularized periosteum‐derived hydrogel (dPH). In comparison with Matrigel, a non‐periosteum‐derived ECM hydrogel product, the dPH group exhibited a significant increase in the expression of osteogenesis‐related genes. This study indicates that dPH has the potential to establish a favorable osteogenic bone microenvironment and can be promising for application in bone defect repair [57]. Porcine small intestinal mucosa provides robust support for cell growth, tissue regeneration, and repair owing to its abundant content of bioactive components such as collagen and fibronectin. It is widely employed in various tissue engineering applications. However, when it comes to bone tissue engineering, relying solely on porcine small intestine mucosa for the treatment of bone defects often proves insufficient in ensuring efficacy. To address this issue, Cheng et al. developed an artificial intestinal mucosa scaffold coated with hydroxyapatite (HAp) and methyl methacrylate gelatin. This innovative approach promotes osteogenic differentiation through the BMP‐2/Smad signaling pathway [58]. The artificial scaffold demonstrated superior osteogenic activity in vitro, resulting in the production of denser, more mature bones compared to pure intestinal submucosal periosteal scaffolds. Amniotic membrane, obtained from the innermost layer of the placenta, offers an alternative to periosteum‐derived dECM and small intestinal submucosa [59]. Ghanmi et al. investigated the efficacy of amniotic membrane as a periosteal substitute in promoting bone regeneration in critical dimensional defects, showing positive results even without natural periosteum [60]. However, the additive effect differed from that of natural periosteum, potentially related to immune responses at distinct sites. Cell‐free human dermis contains fibronectin and hyaluronectin that promote vascular remodeling. Moreover, it can be employed to synthesize biomimetic platforms with capabilities for cell migration and osteoinductive protein delivery. Schonmeyr et al. described a method for synthesizing periosteum‐like materials using acellular human dermis and osteoblasts or MSCs. The study demonstrated the ability of cell‐free human dermis to serve as a tissue‐engineered periosteum capable of delivering cells and bone‐inducing proteins, facilitating the repair of critical‐size bone defects [61]. Beniker et al. obtained new bone formation with minimal soft tissue invasion 6 weeks after implanting acellular dermal periosteal scaffolds into a pig segmental bone defect model [55]. This indicates that dermal membrane materials can serve as scaffolds for periosteal regeneration, supporting cell regeneration, vascular reconstruction, and bone defect repair. These findings suggest that tissue‐derived dECM scaffolds hold potential as substitutes for periosteum, promoting bone regeneration. Cell‐derived dECM scaffolds and cell sheets offer advantages, such as avoiding host immune responses and pathogen transfer [62]. Obtaining cell‐derived dECM involves removing cell components from cell flakes. These dECM variants possess distinct biophysical and biochemical properties based on the cell type employed [63]. Fibroblast‐derived dECM, when inducing re‐inoculation of adipose‐derived MSCs in vitro, positively impacts cartilage differentiation [64]. Preosteoblast‐derived dECM can be employed to develop biomimetic platforms supporting cell adhesion and proliferation, thereby promoting osteogenic differentiation of re‐inoculated MSCs [65]. Combining preosteoblast‐derived dECM with GelMA hydrogel constructs a periosteum‐bone engineering substitute, resulting in accelerated healing of rabbit radius segment bone defects within 12 weeks [66].

MSCs‐derived cell sheets, as prepared by Long et al., exhibit enhanced osteogenesis during critical‐size bone defect repair, showcasing the feasibility of this tissue engineering solution for large‐scale allograft healing. MSCs cell sheets induce prolonged cartilage formation, enhanced bone callus formation at the graft‐host junction, and superior graft‐host bone integration, with two‐cell sheets generally outperforming single‐cell sheet in function [67]. Engineered periosteum composed of sheets of osteoblasts or MSCs exhibits limited vascularization capacity. Kang et al. addressed this by culturing human mesenchymal stem cells (hMSCs) to form cell sheets, inoculating human umbilical vein endothelial cells (HUVECs) onto undifferentiated hMSCs to create vascularization cell sheets [68]. To mimic the fibrous layer of the natural periosteum, mineralized hMSCs were cultured to simulate the cambium of the natural periosteum. They mineralized hMSCs sheet is encased on a cylindrical β‐tricalcium phosphate (β‐TCP) scaffold and further encased in a vascularized HUVEC/hMSC sheet, forming bone substitute on the β‐TCP scaffold. In vivo experiments confirm that this biomimetic platform, resembling the composition and spatial structure of periosteal cells, significantly contributes to angiogenesis and osteogenesis.

The application of cell‐derived dECM scaffolds and cell sheets emerges as a promising strategy for periosteal tissue engineering. While these materials with good biocompatibility promote cell attachment, proliferation, and differentiation, they inherently lack mechanical strength suitable for large defects [69]. Attempts to address this limitation include creating functionally enhanced cell sheets [70]. Structurally enhanced single‐sided or double‐sided cell sheets are prepared through physicochemical modifications and signal responsiveness to increase their structural stability without compromising function [71]. Consequently, future acellular periosteal scaffolds and cell plates should be designed to mimic periosteal structures and incorporate multifunctional features.

Direct delivery of MSCs by biomimetic periosteal scaffolds or cell sheets obtained using decellularized techniques often results in tissue segment separation, poor graft localization, and limited cell survival. To overcome these complications and simulate periosteal function, it is necessary to develop suitable scaffolds to optimally support initial adhesion and subsequent cell growth. Many biocompatible and biodegradable biomaterials have been studied, including natural polymers, synthetic polymers, and combinations of them, in the form of membranes, sponges, hydrogels, etc.

### 4.2 Natural Materials for BP Construction

Collagen membrane demonstrates high biocompatibility, facilitating cell adhesion and proliferation on its surface [72]. Notably, the degradation of collagen membrane does not adversely affect surrounding tissues. Commercially available collagen membranes, such as Bio‐Gide, have been utilized as BP, serving as a biological barrier to promote and guide bone regeneration [73]. However, their extensive use in constructing bionic bone membranes encounters challenges, including low mechanical strength, rapid degradation, poor antimicrobial properties, and the potential transmission of animal‐derived disease. Consequently, these membranes are commonly employed as matrix materials and are often combined with other substances to prepare bionic periosteal constructs. Li et al. modified collagen membranes with bioactive glass to modulate the bone immune response and enhance the osteogenic differentiation of MSCs [74]. This bio‐ceramic bionic composite periosteal scaffold functions as a bone immunomodulator. Inspired by the bone immune microenvironment and the structural function of the natural periosteum, Xu et al. developed a hydrogel‐electrostatically spun biphasic periosteum. In their study, the hydrogel phase of carboxymethyl chitosan collagen (CMC‐Col) cross‐linked on electrostatically spun fibers not only achieved the proliferation, adhesion and differentiation of osteoblasts but also precisely regulated the early inflammatory outbreak. As the hydrogel phase degraded, the synergistic effect of IL‐4 and collagen created a favorable microenvironment for vascular and bone regeneration [75].

Gelatin and GelMA are frequently employed materials in the development of BP, offering excellent biocompatibility and biodegradability. Modifying these materials enhances their degradation rate and facilitates the efficient incorporation of biomolecules and other cell adhesion ligands, thereby expanding their applications in gene networks, cellular networks, and drug delivery [76]. However, the limited bone conductivity due to the absence of osteogenic components or other ions that promote mineralization results in the slow healing of bone defects [77]. Therefore, prior studies have sought to enhance angiogenesis and mineralization by combining BP with bioactive substances to expedite bone regeneration. For instance, Yang et al. engineered a bone paste with adhesive properties by incorporating bioactive glass into a hydrogel crosslinked with oxidized HAp and dopamine‐modified gelatin [78]. This bone paste not only exhibits outstanding adhesive properties, firmly bonding to the surface of the bone defect, but it also has the capability to recruit osteogenic stem cells. Additionally, it promotes osteogenic differentiation and initiates multicentric osteogenesis, thereby accelerating vascularization in the defect area. This synergistic effect contributes to the enhanced repair of bone defects. In another study, Zhang et al. employed mesoporous bioactive glass nanoparticles and GelMA methacrylate organic‐inorganic double cross‐linking technology to construct bionic periosteal scaffolds with varying inorganic ion contents [79]. The results indicated that the organic‐inorganic BP could expedite vascularization in the defect area and coordinate macrophages and stem cells to enhance bone defect repair. Liu et al. fabricated a hybrid hydrogel fiber BP by combining calcium phosphate nanoparticles (CaPs) and GelMA using the electrostatic spinning technique. The integration of CaPs endows the BP with robust mechanical properties and achieves sustained Ca^2+^ release over an extended period [80]. In vitro assessments demonstrated that this BP displayed commendable osteogenic and vasculogenic capabilities.

Chitosan exhibits significant potential in bone repair, attributed to the biocompatibility and biodegradability of its natural polysaccharides, rendering it a material of considerable interest [75]. Notably, chitosan aids in averting immune reactions and stimulates osteoblast proliferation, offering vital support for bone tissue regeneration. Its porous and adaptable structure also positions it as an effective drug delivery carrier, allowing precise modulation of drug release rates [81, 82]. In comparison to collagen materials, chitosan proves cost‐effective and conducive to mass production for clinical applications. However, in simulated body fluid, pure chitosan materials fail to form an apatite layer on their surface, a crucial indicator of osteogenic bioactivity for biomaterials. This limitation underscores its suboptimal in vitro osteogenic bioactivity [83]. Additionally, chitosan faces challenges in terms of mechanical properties, falling short for bone paste applications [84]. Therefore, enhancing its bioactivity and mechanical strength becomes imperative to foster mineralization and early bone regeneration.

Recent studies have incorporated various bioactivity enhancement methods with chitosan‐based membranes to improve bone conductivity and bioactivity. Bhushan et al. manufactured a composite BP (CG‐CNPs) using chitosan, gelatin, and cerium oxide nanoparticles, exhibiting superior physicochemical, mechanical, and biological properties [85]. It also displayed a broad inhibitory zone against both gram‐positive and gram‐negative bacteria, showcasing its potential for bone defect repair. Renata et al., through chemical modification of chitosan, used phosphorylated polymer to produce scaffoldings based on chitosan‐xanthan gum, demonstrating heightened bone inducibility, with the material concentrating natural BMP and inducing bone formation when used in vivo [86].

Furthermore, alginate is a common material in the development of bone tissue engineering scaffolds. Zhou et al. introduced an adaptive bionic periosteal strategy—an interpenetrating dual‐network hydrogel composed of diselene‐containing gelatin and calcium alginate [45]. This novel hydrogel enhances periosteal stability, exhibits anti‐swelling properties, and delays degradation. The adaptive BP with interpenetrating double network structures has proven safe and effective in repairing critical size bone defects. Additionally, based on chitosan membrane, a chitosan‐tricalcium phosphate‐gelatin scaffold can be constructed as a tissue‐engineered periosteum with enhanced mechanical properties [87]. The use of a tissue‐engineered periosteum based on chitosan‐heparin membrane, locally carrying fibroblast growth factor‐2 (FGF‐2), transforming growth factor‐β1 (TGF‐β1), and adipose mesenchymal stem cells (AMSCs) to repair femoral defects in mice, has also been evaluated [88]. Beyond these natural components, cellulose and other materials have been considered for their potential to create biomimetic periosteal materials. However, naturally sourced polymers may exhibit poor mechanical properties and harbor pathogenic impurities, limiting their clinical application.

### 4.3 Synthetic Materials for BP Construction

In comparison to natural polymeric materials, synthetic polymers exhibit highly controllable characteristics, superior mechanical performance, degradability, strong processability, and cost‐effectiveness. These attributes have garnered significant attention for synthetic polymers in the field of BP. The synthetic polymers most commonly utilized in BP development include polycaprolactone (PCL), poly(L‐lactic acid) (PLLA), polylactic‐glycolic acid copolymer (PLGA), polyurethane (PU), poly[(R)‐3‐hydroxybutyric acid] (PHB) and polyethylene glycol (PEG) [50, 89–92]. While these polymers exhibit good compatibility, they lack sufficient guidance for bone cells when applied in BP. This deficiency significantly hinders the proliferation and differentiation of bone cells on their surfaces, thereby greatly limiting their effectiveness in bone repair. Additionally, the intermediate degradation products of these polymers may potentially trigger inflammation and immune reactions, introducing considerable uncertainties into the bone repair process [96, 97]. Therefore, current synthetic polymers often incorporate various bioactive components, such as magnesium oxide (MgO) or ECM, to construct composite BP [52].

PCL is a semi‐crystalline aliphatic polymer that demonstrates good biocompatibility, adjustable biodegradability, excellent mechanical properties, and high customizability [98]. Moreover, PCL demonstrates stability in degradation and features a prolonged degradation cycle, rendering it a favorable option for bionic periosteal applications. Despite obtaining U.S. Food and Drug Administration (FDA) approval for clinical use, challenges persist in bionic periosteal medical applications due to PCL's hydrophobic nature, impeding cell proliferation and adhesion [99]. Additionally, it lacks components conducive to the promotion of osteogenic differentiation. Wang et al. implanted composite nanofiber sheets comprising PCL, collagen, and nano‐hydroxyapatite (nHAp) into the bone marrow matrix [100]. A bottom‐up layer‐by‐layer strategy was employed to construct BP. When used in conjunction with structural bone allografts to repair segmental bone defects in the mouse femur, BP successfully reinstated bone formation in the periosteum at the donor site. This reversal of biomechanics addressed the challenge of poor healing observed in allograft bone after implantation. Current research is focused on developing hierarchical BP structures that emulate the natural periosteal architecture and encompass multifunctional features [101]. Sun et al. employed stepwise conjugated electrospinning technology to construct BPs with a three‐layer interfacial structure. These BPs closely emulate the structural and functional characteristics of natural periosteum. The mechanical properties exhibit a gradual decrease from the inner to the outer layers, while degradation and cell permeation properties exhibit an increase in the same direction. Furthermore, the osteogenic properties of the composite membrane were enhanced through the incorporation of HAp [95].

PLLA is an aliphatic polyester renowned for its biocompatibility and exceptional mechanical properties. It has garnered FDA approval for diverse biomedical applications in clinical implants. However, akin to PCL, PLLA exhibits inherent limitations, including hydrophobicity, biological inertness, and the adverse impact of acidic degradation products on osteoblast adhesion and proliferation [102]. Consequently, researchers are actively engaged in mitigating these constraints through diverse strategies. These encompass surface modifications, copolymerization with alternative materials, and the formulation of composites that amalgamate PLLA with substances to compensate for its limitations [103, 104, 105]. Wu et al. introduced a BP consisting of a layered micro/nano‐fiber structure [92]. The BP created through collagen self‐assembly and micro‐solvent electrospinning possesses a hierarchical micro/nanostructure that aptly mimics the characteristics of periosteum. The cambium, serving as an imitation of the extracellular matrix, plays a pivotal role in cellular processes. Furthermore, the study incorporated a hyaluronic acid (HA)‐PLLA core‐shell structure to encapsulate VEGF. This configuration enables the sustained and controlled release of VEGF, promoting angiogenesis within fibrous layers and areas of bone defects. The engineered periosteum thus demonstrates promising capabilities for creating a conducive environment for tissue regeneration. Hua et al. employed coaxial electrostatic spinning technology to manufacture nanofiber membranes utilizing PLLA/HA [106]. The incorporation of irisin into these fiber structures facilitated the controlled release of irisin. This composite BP not only exhibits commendable bioactivity on its own but also addresses the limited osteogenic capacity of PLLA by incorporating these natural active ingredients, thereby enhancing its overall performance.

The piezoelectric properties of PLLA are intriguingly gaining prominence in the realm of tissue repair. Within its structure lies a flexible helical chain comprising C═O dipoles [107]. Ordinarily, PLLA exhibits no significant piezoelectric effect. Nevertheless, through processes such as high‐temperature annealing, stretching, and exposure to electric fields, the crystal structure of PLLA can be altered to manifest piezoelectricity. Presently, the piezoelectric constants of PLLA span from 9 to 19 pC/N [108]. Although there are no current reports on PLLA piezoelectric periosteum, previous studies have explored PLLA piezoelectric films or scaffolds for bone tissue repair in other areas. In a study by Liu et al., a biodegradable scaffold was developed using PLLA nanofibers through electrostatic spinning [109]. These nanofibers exhibited piezoelectricity under external forces, enhancing chondrogenesis in vitro. Rabbits with osteochondral defects treated with PLLA scaffolds showed improved cartilage and subchondral bone regeneration due to piezoelectric charges generated during joint loading exercises after 1 or 2 months. This study suggests that biodegradable PLLA piezoelectric scaffolds could be applied in osteoarthritis treatment with joint‐loading exercises. In the application of BP, the nanofiber with a highly oriented structure, crafted by them, not only mimics the structure of natural periosteum but also has the capability to generate piezoelectric charge through movement for therapeutic purposes. Additionally, the therapeutic method of generating piezoelectric charge through movement is a subject worth researching and exploring. Zheng et al. introduced various volume fractions of calcium‐manganese co‐doped BaTiO_3_ (CMBT) into PLLA fibers to enhance their piezoelectric properties [110]. This improvement, along with the release of bioactive ions, contributed to enhanced osteogenic properties. Significantly, the therapeutic efficacy of this biodegradable piezoelectric film surpassed that of typical biodegradable synthetic materials, addressing the challenge of balancing high piezoelectricity with degradability. Consequently, we posit that PLLA membranes with piezoelectric properties hold significant potential for bionic bone membrane construction.

Among synthetic materials, PLGA stands out as one of the most frequently employed substances in biomedical applications, including surgical suture materials, and it has received approval from FDA [111, 112]. Research has demonstrated that PLGA fibers play a crucial role in facilitating the formation of multilayers that accurately mimic the highly organized ECM present in the periosteum, particularly in the context of bone graft repair and reconstruction. To overcome their deficiency in osteoinductivity and hydrophobicity, various technologies have been developed. In a study conducted by He et al., BP was created through electrospinning using PLGA/MgO/quercetin [99]. The composite membrane exhibits a highly porous microstructure, creating a favorable surface morphology for cell adhesion. Additionally, the presence of magnesium ions (Mg^2+^) and a quercetin dose activates the Wnt/β‐linker pathway, facilitating vascular and bone ontogeny.

In addition, some non‐biodegradable synthetic polymers such as conductive polyvinylidene fluoride (PVDF) with special piezoelectric properties and poly(vinylidene fluoride‐trifluoroethylene) [P(VDF‐TrFE)] membranes [113, 114]. Wu et al. utilized electrostatic spinning to create non‐invasive microenvironments, electrically stimulated and reminiscent of periosteum [115]. These environments consist of both disordered and highly oriented PVDF nanofibers. Through annealing, the materials acquired enhanced piezoelectric properties. Their research uncovered that PVDF membranes, influenced by a significant piezoelectric effect, influenced stem cell fate and facilitated more active calcium transfer. Triggering the piezoelectric effect on the PVDF film with high voltage led to increased cell adhesion area, a stable microenvironment, and heightened calcium metastasis. Consequently, this promoted superior differentiation of BMSCs. The study introduces a novel perspective for exploring intricate interactions within the periosteal microenvironment. Moreover, these findings establish a foundational understanding for designing BP. In another study, Zhang et al. designed PVDF grooves with piezoelectric nano‐topography [116]. This unique nanostructure, along with the piezoelectric effect of PVDF itself, not only promotes the proliferation and adhesion of rat BMSCs but also induces surface motion and traction on the membrane, generating a surface piezoelectric potential of up to millivolts. This piezoelectric potential can spontaneously promote the differentiation of BMSCs into neuron‐like cells without the need for external electric fields or neurotrophic factors. This study not only provides a new understanding of piezoelectric materials for the next generation of neural engineering but also offers new methods for constructing bio‐inspired periosteum with neurogenic capabilities. The biologically inert nature of PVDF requires a secondary procedure for removal post‐implantation, leading to concerns such as patient discomfort, surgical expenses, and infection risk. Achieving a balance between the piezoelectric properties and degradability poses a challenge in applying inert piezoelectric materials in the BP field.

To sum up, in tissue engineering and regenerative medicine, biomaterials provide mechanical support and biochemical signals to encourage cell attachment and regulate cell behavior. Generally, the natural template for biomaterials is the ECM, which contains intrinsic biochemical and mechanical cues that regulate cell phenotype and development, homeostasis in vivo, and response to injury. The application of ECM‐based materials in biomedical research has ranged from coating cell culture plates with purified ECM components to the design of biomaterials mimicking ECM and acellular tissue engineering aimed at outlining the dynamics, composition, and structure [117]. dECM‐derived BP has the best biocompatibility, which can promote cell attachment, proliferation, and differentiation, as well as provide a barrier for bone defect repair. In addition, because it is derived from acellular tissues or extracellular matrix of cells, it perfectly simulates the naturally existing cell niche, and dECM materials are in a sense the best choice for BP construction. However, its extremely low mechanical strength limits its application in large bone defects. And there are potential implantation risks including tissue segment separation, poor graft localization, and limited cell survival [62]. Natural polymers are highly biocompatible and can also support the attachment and proliferation of cells on their surfaces. Notably, their degradation products generally have no adverse effects on surrounding tissues. However, natural polymers have poor mechanical strength and a fast degradation rate, requiring specific modifications to increase their mechanical properties and reduce their degradation rate [78, 79]. Synthetic polymers own suitable biocompatibility, better mechanical properties, and more transformable properties that can be joined, and have gradually become a hot material for artificial tissue engineering periosteum. However, the biological activity of some synthetic polymers is insufficient, which requires researchers to carefully design and combine them with bioactive factors or ions for optimal function. In addition, the degradation products of some polymers are also toxic, limiting their clinical application [108, 109]. Therefore, it is particularly difficult to combine various biological materials to prepare BP with well biocompatibility, suitable mechanical strength and sufficient biological activity. This requires that the manufacturing techniques involved must be sufficiently advanced to satisfy the construction of the ideal BP.

### 4.4 General Techniques for BP Construction

Natural and synthetic materials exhibit excellent control over mechanical or degradation properties. However, both of them encounter challenges in terms of biocompatibility, bone conductivity, and bone induction. Consequently, there is a significant demand for further refinement of natural and synthetic materials to design enhanced bioactive BP for clinical applications. Currently, techniques for constructing BP include solvent casting, hydrogels crosslinking, electrostatic spinning, and 3/4D printing (Table 2).

**TABLE 2:** Comparison of general strategies for constructing BP.

Technique	Advantages	Disadvantages	Ref.
Solvent casting	Simple, low‐cost, easy to prepare BPs with specific shapes and porous structures	It is difficult to construct BPs with complex 3D structures and multi‐layer features, solvent residues may affect biocompatibility	[132]
Hydrogel crosslinking	Well‐biocompatibility and ability to mimic natural extracellular matrix, support cell proliferation and migration	Uncrosslinked hydrogels have poor mechanical properties, but the crosslinking process may affect bioactivity. It is challenging to control the crosslinking degree and degradation rat.	[78]
Electrostatic spinning	Allow BPs owing highly customized fiber structures and porosities, mimicking natural periosteal fiber structure	Relatively low fiber yield, high demands on operating conditions such as voltage and flow rate, limited controllability of fiber diameter and distribution	[137]
3D/4D printing	Precise control over the microstructure and macroscopic shape of BPs, suitable for complex and personalized BP designs	High equipment cost, relatively slow printing speed, and high requirements for material processability. Time response control in 4D printing needs further research and development	[115, 139]

The solvent casting method (SCM) is a straightforward process involving the dissolution of film material in an appropriate solvent This material is then formulated into a uniform solution and poured onto a flat glass plate or Petri dish. Utilizing a specialized spatula, the solution is evenly spread to form a thin, uniform film. Subsequently, the film is transferred to a well‐ventilated area to facilitate solvent evaporation, ultimately resulting in the creation of a uniform film [132]. This technology allows for the preparation of porous films not only by exploiting the effects of phase separation, but also by customizing molds with specific shapes to prepare film morphologies that are beneficial for stem cell proliferation and differentiation. Nevertheless, constructing bionic structures with periosteal multilayer features using SCM remains a challenging endeavor. Shi et al. pioneered the development of PLGA nanosheets featuring grooved micropatterns to emulate natural periosteum for use in bone repair therapies. These nanosheets exhibit excellent adhesion to bone grafts in aqueous conditions, maintaining stability on the surface of the grafts. The distinctive groove structure also serves to modulate surface‐associated stem cells, enhancing the osteogenesis of the membrane [50].

In contrast to thin film structures prepared by SCM, the internal structure of hydrogels closely resembles that of natural periosteal connective tissue. This similarity allows hydrogels to offer three‐dimensional support for cell proliferation and tissue generation. Typically, hydrogels blended with various bioactive substances facilitate angiogenesis and bone regeneration through immunomodulation [133]. However, the mechanical properties of most hydrogel‐based BPs often tend to be poor and struggle to meet the mechanical requirements of periosteal structures. Traditionally, enhancing mechanical properties involves introducing physicochemical cross‐linking in the hydrogel network or incorporating inorganic nanofillers. For instance, Ullah et al., a hydrogel BP was designed and crosslinked using a hybrid inorganic/organic crosslinking approach [134]. The researchers enhanced the osteogenic differentiation capacity of the BP by introducing metal ions strontium (Sr) and/or iron (Fe) into HAp. This modification with nanoparticles in the photocrosslinked methacrylate GelMA not only improved degradability and mechanical properties but also ensured pH stability in the microenvironment of BP degradation. The incorporation of inorganic nanoparticles to enhance the overall performance of hydrogels is a well‐established and widely employed strategy in the construction of contemporary BPs. In a recent study, Chen et al. incorporated polyvinyl alcohol (PVA) hydrogels containing curcumin and phytic acid (PA) into delignified wood to fabricate wood hydrogels with anisotropic and robust mechanical properties. Moreover, this hydrogel exhibits synergistic effects in inhibiting bacterial growth and inflammatory responses while promoting osteogenic differentiation [135]. The approach of enhancing mechanical properties through a templating method and imparting additional biological functions to the BP via the inclusion of natural active substances proves to be a valuable strategy.

Electrostatic spinning proves to be an effective method for fabricating nanofibers from polymers in the preparation of BP. The drum speed adjustment allows for the creation of a gradient bionic structure with similarity to natural periosteum. Furthermore, the advantages of electrospinning encompass controllable fiber size and porosity, facile fabrication, and low manufacturing cost [136]. Existing studies have demonstrated the advantages of nanofibers with an oriented topology constructed under a drum rotating at high speeds, typically 3000 rpm or more. First, this structure serves as an effective barrier, preventing the growth of surrounding soft tissues into the bone defect site. Second, it can stimulate macrophages to transition from M1 to M2 phenotype, leading to increased secretion of anti‐inflammatory cytokines and pro‐healing cytokines, thereby creating a favorable microenvironment for bone defect repair [137]. We believe that it is promising to utilize this structure in conjunction with other techniques in the future preparation of electrostatically spun periosteum.

Finally, the precise arrangement of cells and scaffolds by 3D or 4D printing allows two layers of periosteum with strong adhesion to be fixed on the bone surface, or even directly made into an integrated bone‐periosteum structure. 4D printing can be simplified as “3D printing + time,” wherein 3D printed components undergo changes over time in response to external stimuli (such as heat, magnetism, light, humidity, pH, etc.), affecting shape, performance, and function [138]. Therefore, 4D printing technology emerges as a valuable tool for achieving structural and functional integration in BP. Liu et al. pioneered the construction of personalized BP with anisotropic microstructure by introducing an adjustable shape structure to maintain the bone remodeling microenvironment [115]. Through 4D printing technology, they systematically assembled cell sheets on a deformable hydrogel containing both biophysical signals and adjustable spatial characteristics. The outer hydrogel layer of the BP allows digital adjustments to its three‐dimensional geometry, aligning with the macroscopic bone shape and simultaneously safeguarding the bone healing microenvironment. The inner layer of hMSCs within the BP not only enhanced co‐culture cell migration and vascularization but also demonstrated outstanding osteogenic differentiation capabilities. In vivo experiments confirmed that this systematically arranged BP not only acted as a physical barrier for shape changes but also actively promoted local vascularization and early‐stage bone formation. You et al. fabricated a bilayer deformable film using 4D printing [131]. The responsive surface microstructure of the shape memory polymer(SMP) layer allows precise adjustments during proliferation and differentiation stages, fostering bone formation. The hydrogel layer equips the BP with the ability to digitally adjust its three‐dimensional geometry to match specific macroscopic bone shapes. In vivo studies revealed that the 4D deformable film exhibited over 30% improvement in new bone formation compared to reference membranes with static microstructures. Moreover, the 4D film could non‐invasively encapsulate bone defect models, providing a novel strategy for addressing complex tissue defects.

In future studies, we believe that the preparative means of constructing BP should not be limited to a single technical means. The concatenation of multiple techniques to complement each other may be a hot spot in the future. For example, combining the advantages of hydrogel with traditional SCM to mimic the different layers of the periosteum structure. At the same time, the different biological requirements of the upper and lower layers of the periosteum structure can also be used to construct Janus BP with special properties, which is a very interesting research direction.

## 5 BP Construction for Structural Biomimicry

Tissue engineering periosteum with biomimetic structures, such as monolayer (hydrogel base membrane, spun fiber membrane) and multilayer (Janus structure, sandwich layer structure, double dam structure) are summarized in this section according to different biomimetic structure modes and preparation techniques. Structural biomimicry demonstrates two levels including the macrostructure involving single‐layered, double‐layered and multilayered structure, as well as the microstructure of surface embracing micro‐groove, micro‐pattern and micro/nano morphology.

### 5.1 Monolayer Structure

Monolayers are typically fabricated from a substrate material such as hydrogel base membrane, spun fiber membrane, organic matrix membrane et al, subsequently processed mainly through a preparation technique, and often serve as the building blocks for multilayer films. Although monolayer membranes have a relatively simple function, reproducing the versatility of the periosteum often requires combining biochemical and physiological factors. Hydrogels, characterized by a strong hydrophilic three‐dimensional network structure, simulate periosteal ECM effectively by facilitating nutrient transport and waste removal. Natural polymers like GelMA methacrylate, rich in Arg‐Gly‐Asp (RGD) sequences promoting cell adhesion, form a single‐layer structure covering the bone injury area, aiding in the bone repair process. However, GelMA's excellent hydrophilicity poses challenges for its slow‐release functionality. Sun et al. proposed an artificial periosteum with directional surface nano‐morphology and robust bone adhesion [140]. They employed an inverse opal to stretch the PLA membrane, generating a directional elliptic structure. Carbon nanotubes coated on the membrane surface acted as a bio‐simulator of extracellular matrix fibrin. The high tensile flexibility of carbon nanotubes ensured membrane integrity during stretching, leading to a collagen fiber‐like orientation structure that induced cell arrangement after seeding. Polydopamine (PDA) hydrogels at both ends of the membrane, inspired by mussels, facilitated bio‐adhesion to diseased tissue. The integration of these functional materials resulted in a new artificial periosteum meeting the requirements of the entire bone repair process. Electrospinning, a polymer fluid electro atomization process, is a suitable choice for artificial periosteum preparation. Lu et al. utilized electrospinning to prepare porous PLLA fiber monolayer for bone tissue engineering substrates. Silica nanoparticles coating owning expansive surface area and excellent biocompatibility can not only enhance the mechanical strength and hydrophilicity of the membrane but also improve the adhesion and cell proliferation ability [141]. Polyether ether ketone (PEEK), known for its elastic modulus matching natural bone cortex strength, was prepared into nanofibers by Zhao et al. using electrostatic spinning. They combined PCL and sulfonated nanofibers to create a soft composite periosteum (s‐PEEK/PCL) that is more hydrophilic, more malleable, more bioactive, and more capable of protein adsorption [142]. Therefore, the single‐layer composite membrane demonstrates high osteogenic potential.

Bone microenvironment and bone defect repair are highly dynamic, including various mechanical properties and metabolic functions. This mechanical strengthening and metabolic absorption is a process of bone remodeling [143]. Bone tissue is mainly composed of type I organic collagen and inorganic mineral carbonated HAp. When minerals such as HAp are deposited into the collagen fiber inter substance, collagen mineralization occurs and carbonized HAp crystals nucleate outside the collagen fibril, resulting in both internal and external mineralization of collagen fibrin, which may be included in the bone‐like template [144]. Previously, people used type 1 collagen as an organic substrate and found that its disadvantages were very obvious, such as high biodegradability and low mechanical strength. In addition, collagen self‐assembly is difficult in vitro, which may result in a low‐density membrane network, resulting in poor tissue structure. Therefore, the composition structure of biomimetic organic matrix source is also a necessary strategy. Recently, Sandra Hofmann's team developed a collagen mineralization technique to mineralize silk fibroin (SF) membranes [145]. The adopted poly aspartic acid (pAsp) as an alternative to mineralizing solutions and integration into SF materials to improve its mineralization. SF acts as an organic matrix and pAsp can mimic the function of non‐collagen proteins. First, pAsp was added to the mineralization solution to mineralize the SF material internally and acted as a coating for the SF film. This mineralized organic matrix membrane promotes osteoclast uptake and osteoblast mineralization in specific sequences, as well as physiologic bone reconstruction. In addition, a single layer of artificial periosteum was prepared from tussah silk protein (AF) membrane by biomineralization technology. After mineralization, its mechanical property is strengthened, showing advanced elastic modulus and tensile strength. The osteogenic differentiation of MSCs was significantly promoted in vitro without the need of osteogenic inducers [146].

### 5.2 Surface Microstructure

**FIGURE 3:**
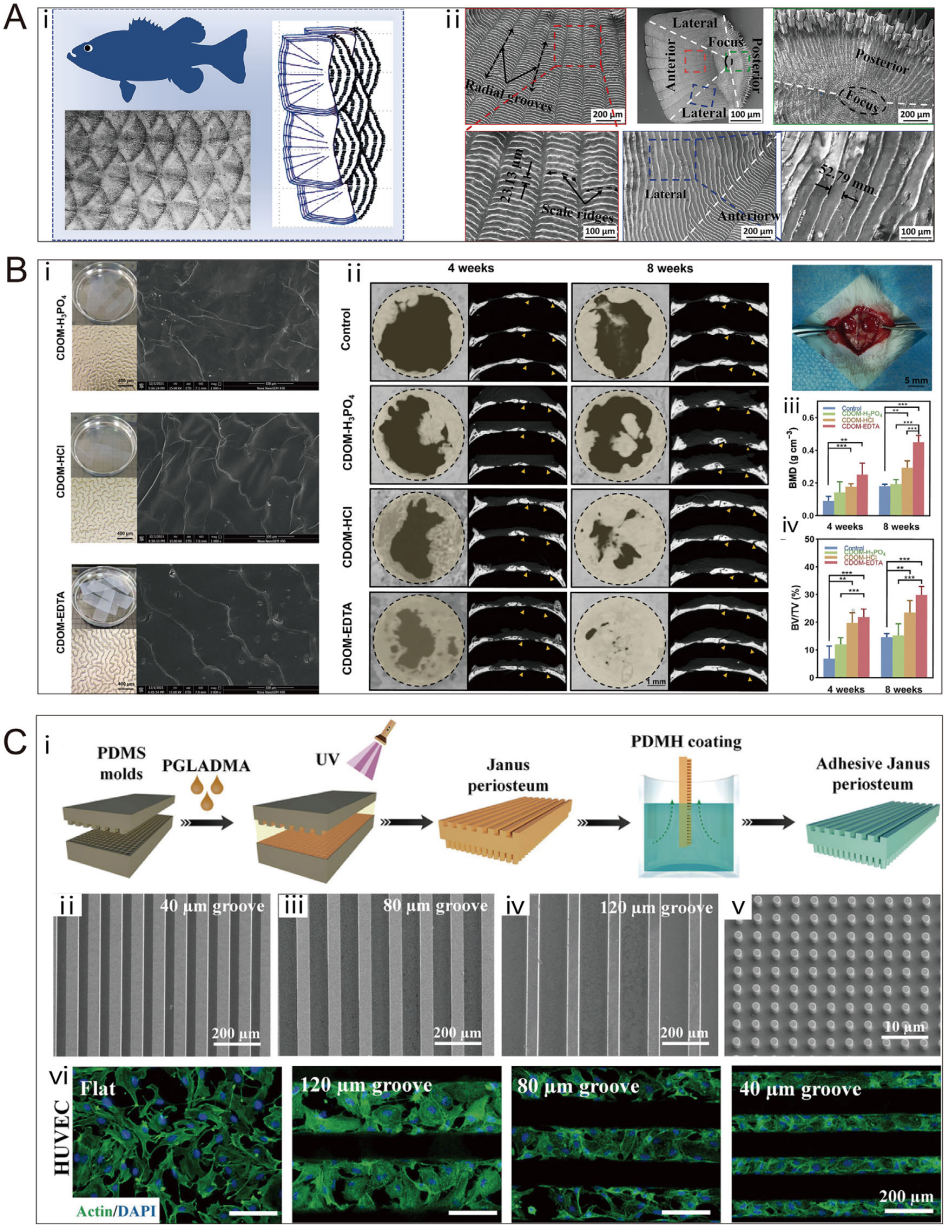
BP with surface microstructure. (A) Morphological and structural characterization of the scales of Lateolabrax japonicus. (i) Figure discusses the morphological and structural examination of fish‐like scales on Lateolabrax japonicus. (ii) SEM image that details the micropatterned surface of these scales post‐decellularization, illustrating the intricacy of their outer structure. Reproduced with permission [147]. Copyright 2023, Elsevier. (B) Morphology and in vivo therapeutic effects of cuttlefish bone periosteum. (i) Optical microscope and scanning electron microscope photographs for characterization of CDOM films. (ii) 3D reconstructed micro‐CT images illustrate new bone growth at 4 and 8 weeks following surgery. (iii) Representative photographs of the established model of a critical‐sized cranial defect. (iv) New bone formation at 4 and 8 weeks postoperatively. Quantitative statistics of BMD and (v) BV/TV. BMD: bone mineral density; BV/TV: bone volume/total volume. **p < 0.01, ***p < 0.001. Reproduced with permission [148]. Copyright 2023. (C) Manufacturing and characterization process of an adhesive BP. (i) Schematic depicting the fabrication of Janus periosteum by photocrosslinking—assisted injection molding technique. SEM images of the adhesive BP. including microgroove‐patterned surfaces with different microgroove widths on the adhesive surfaces of (ii) 40, (iii) 80 and (iv) 120 µm and (v) fibrous arrays, respectively. (vi) HUVECs on the periosteum mimicking microgroove patterns using actin/DAPI staining on day 3. Green and blue fluorescence indicate cell filaments and nuclei, respectively. Reproduced with permission [149]. Copyright 2019, Wiley.

The periosteum features a structured surface with vertically oriented cells and collagen fibers, influencing cell arrangement, collagen fiber organization, and bone growth direction. Micro‐morphology and nano‐morphology represent a specific functionalization of the bionic periosteal structure, showcasing a nuanced single‐layer membrane with essential bionic structure functions. Qin et al. proposed a morphological strategy to enhance bone regeneration employing the surface micromorphology of Lateolabrax japonicus scales as a physical cue for programming cell behavior [147]. Fish scales are thought to be fish excrement, and their surfaces show rich anisotropic ridged micropatterns (Figure 3A), whose composition is similar to that of natural bones. They prepared acellular fish scales and degelatinized ones from fresh fish scales by decellularization and degelatinization procedures respectively. The anisotropic ridge micropattern surface and mineralized collagen co‐program cell orientation and induce macrophages polarizating into M2 phenotype, which can regulate the bone immune microenvironment by facilitating anti‐inflammatory cytokine secretion. In addition, unique surface morphology and mineralized collagen may synergistically promote BMSCs osteogenic differentiation through the Wnt/β‐catenin pathway. In vivo, the organic matrix of fish scales conspicuously enhanced the proportion of M2/M1 macrophages, upregulated the expression of anti‐inflammatory and pro‐healing cytokines, and promoted osteogenesis and bone repair. Wei et al. obtained a cuttlebone‐derived organic matrix (CDOM) membrane by etching calcium carbonate [148]. According to the macroscopic image of the CDOM and SEM characterization (Figure 3B), the membrane owning a similar surface structure showing an “S” groove above, and a distance of 87±12 um between adjacent columns, which can provide a room suitable for cell proliferation. The opposite side of the membrane is smoother than the front, and there are nanopores on both sides for small molecules to transport. CDOM is composed of polysaccharide and protein, and has an “S” shaped groove in structure, which increases roughness and hydrophilicity, as well as being conducive to cell adhesion during healing, thus exhibiting the capacity of promoting osteogenesis and angiogenesis. Significantly larger new bone areas were observed in the DOM‐EDTA group compared to the control group at 4/8 weeks post‐implantation of cranial defects. Additionally, the CDOM‐HCl group exhibited significant new bone regeneration at the defect site. Notably, the CDOM‐EDTA group demonstrated the highest values for bone mineral density (BMD) and bone tissue volume/total tissue volume (BV/TV). These findings suggest that the “S” grooves present in the CDOM‐EDTA film, along with its unique composition, play a pivotal role in accelerating bone regeneration.

As previously mention, Shi et al. innovatively designed a PLGA nanosheet with an oriented microgroove structure [50]. This structure matched the natural periosteum's ability to modulate cell arrangement, enhancing the robustness of tissue engineering scaffolds. The integration of the oriented microgroove structure with paraffin film, known for its flexibility, allowed the regulation of cell growth direction and mechanical tension application. The addition of a polydopamine coating improved biocompatibility and increased cell attachment and proliferation. Under mechanical stretching and spatial structure induction, AMSCs in the stretched group exhibited superior osteogenic ability, confirming the positive impact of mechanical stress on bone regeneration and osteogenic differentiation. Jing et al. delved deeper into the mechanisms facilitating the enhancement of osteoblast differentiation using groove patterns characterized by varying widths (0.35–7 µm) and depths (0.65–6 µm). The findings unveiled an association between the groove patterns that augment osteoblast differentiation and the cells' capacity to elongate [149]. Moreover, these patterns demonstrated the ability to regulate osteoblast differentiation by suppressing the generation of reactive oxygen species (ROS). This mechanism effectively overcame the inflammatory impediments to osteoblast differentiation. Exploring whether this micrometer notch enhances both osteogenesis and angiogenesis is valuable. Yang et al. designed a Janus structure for the periosteum, featuring different micrometer grooves in the upper layer and an adhesive fiber array inspired by gecko feet in the lower layer (Figure 3C). This Janus BP not only firmly adheres to the surface of bone defects in both dry and wet states but also effectively modulates cellular responses, promoting synchronized osteogenesis and angiogenesis in vitro and in vivo without the addition of any active factors [150]. The development of this Janus bionic osteochondral membranes holds significant guidance for future advancements in BP construction.

In addition to groove structures, innovative biomimetic periosteal membranes feature micro‐patterned morphologies. Withal mineralized hydroxyapatite nanoparticles (HANPs) and hydrogels were utilized to fabricate a bionic artificial periosteal material with a specific micropattern structure, aiming to promote bone healing [151]. Employing a biomimetic mineralization strategy, the uniformly dispersed lamellar HANPs were completed. Micro‐contact printing was then used to create four distinct HANPs micropatterns on polydimethylsiloxane (PDMS) substrates. The subsequent transfer of the HANPs pattern to a 1mm‐thick gelatin film resulted in straight, grid, round, and square micropatterns, confirmed by SEM and fluorescence microscopy. Cells cultured on these patterned biomimetic membranes expressed higher levels of osteogenic and angiogenic markers compared to those on membranes that are not patterned. Moreover, rat BMSCs in the patterned group exhibited enhanced calcium nodule formation, indicating improved cell differentiation. The biomimetic membranes with HANPs micropatterns, coupled with site‐specific mineralization, offer sustained induction of stem cell behavior and differentiation, providing a platform for the controlled release of growth factors. This bionic membrane mimics intramembrane ossification, fostering a microenvironment conducive to angiogenesis, osteogenesis, cell recruitment, differentiation regulation, vascularization, and bone regeneration. The bionic membrane promoted angiogenesis and osteogenesis in rat BMSCs, as demonstrated by highly oriented tissue formation. In a rat skull defect model, the bionic membrane with a biomineralization mode significantly enhanced vascularization ossification and new bone formation. These biomimetic membranes, with specific biomineralized micropatterns, present a promising alternative to autogenous periosteal transplantation, holding substantial potential for clinical applications.

### 5.3 Janus Structure

Natural periosteum has an outer fibrous layer composed of parallel fibrous tissue, and an inner cambium containing various cells and disordered nanofibers. Therefore, the Janus membrane with a double‐layer structure shows different properties on both sides, which can fulfill the dual function of osteogenesis/barrier to guide bone regeneration. A novel biodegradable Janus biofilm was prepared by Lv et al. based on the heat‐sensitive carboxymethyl chitin, which has an asymmetric pore structure [152]. Next, nHAp cost on the single membrane to obtain composite carboxymethyl chitin/nHAp Janus biofilm. It exhibited dual bio‐functions: the dense layer of Janus membranes can act as a barrier to prevent connective tissue cells from invading bone defects, while the porous layer containing nanoparticles (pore size 100–200 µm)‐nHAp can guide bone regeneration. In addition, the ideal BP should have a heterogeneous structure composed of different layers to meet specific requirements. Therefore, Li et al. designed two different arrangements of collagen membranes. One oriented arrangement of collagen was used to simulate the fiber layer of the natural periosteum, which could promote the macrophage transformation of M1 phenotype into M2 one. The other was a randomly oriented collagen used to simulate the cambium layer, which could accelerate osteogenic differentiation of endogenous bone progenitor cells [153].

**FIGURE 4:**
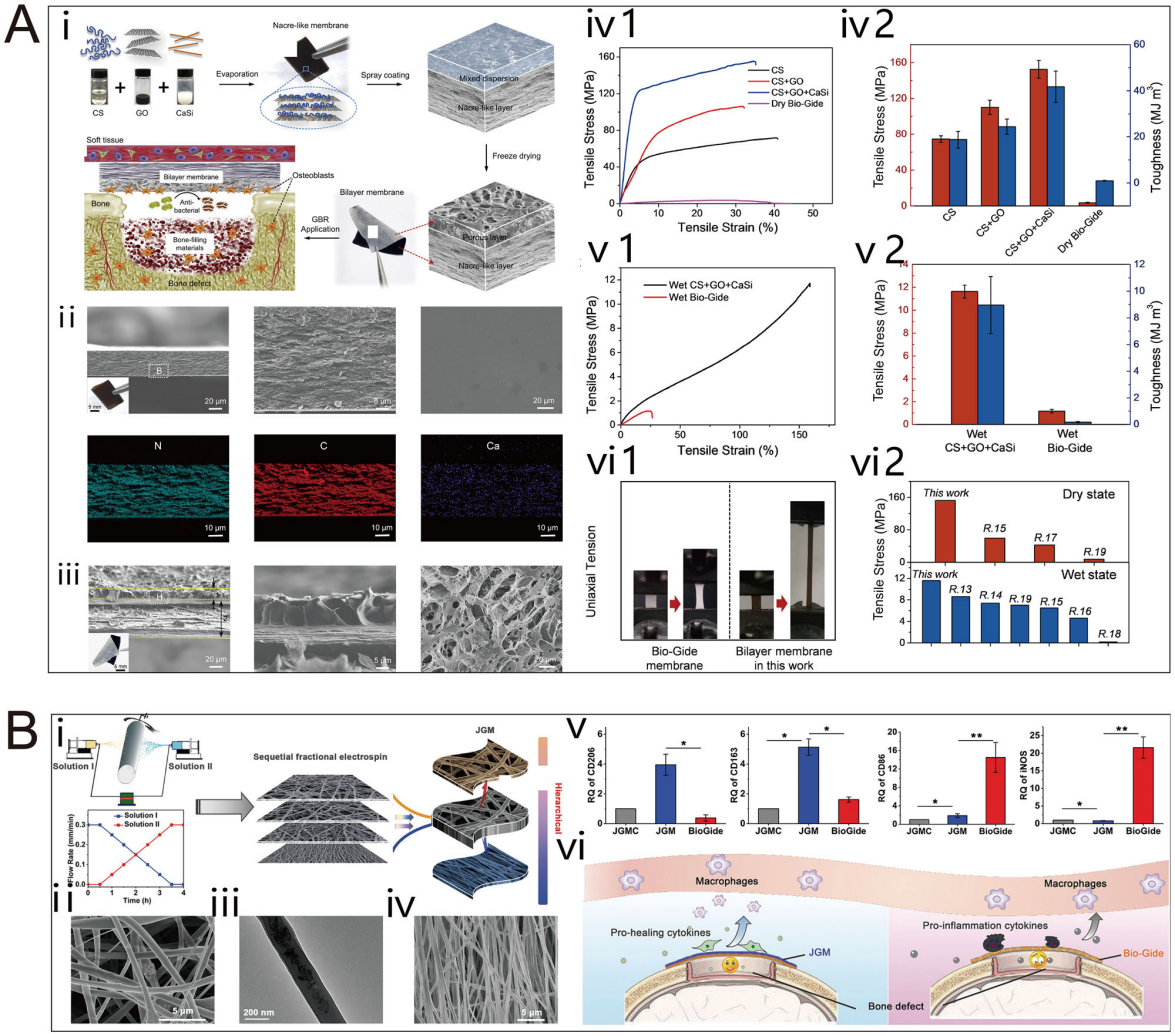
Composite membrane with Janus structure. (A) Preparation process, morphology and mechanical properties of bone‐guided regenerative membranes with shell layer structure and porous composite structure. (i) Preparation of bilayer membrane with shell layer structure and porous structure. (ii) Cross‐sectional and surface morphology of the outer surface of the composite film and its elemental analysis. (iii) Cross‐sectional morphology of the composite film and its inner surface. (iv1, iv2) Typical mechanical curves, tensile strength, and toughness of composite films in the dry state. (v1, v2) Typical mechanical profiles, tensile strength and toughness of composite films in the wet state. (vi1, vi2) Photographs of mechanical tensile in dry and wet conditions. Comparison of tensile properties and toughness of currently published bone‐guided regeneration membranes. Reproduced with permission [154]. Copyright 2019, Elsevier. (B) Characterization of Janus membrane. (i) Schematic diagram of sequential segmented electrospinning process. (ii–iv) Scanning electron microscope images of the inner surface of Janus membrane, TEM images of nanofibers, and scanning electron microscope images of the outer surface of Janus membrane, respectively. The scales of the image scale are 5 µm, 200 nm, and 5 µm, respectively. (v) RT‐PCR for M2 phenotypes (CD206 and CD163) and M1 phenotypes (CD86 and iNOS) and gene expression. (vi) Schematic of the bone healing mechanism. Reproduced with permission [155]. Copyright 2019, Wiley.

Zhang et al. reported a new double‐layer membrane inspired by a natural matrix of Pearl [154]. It was made by combining evaporation‐induced self‐assembly with a subsequent ice template procedure, applying chondroitin sulfate (CS), graphene oxide (GO), and a CaSi nanowire as building blocks, including a dense nacreous layer and a porous layer (Figure 4A). The dense pearl layer imparts strong mechanical properties to the membrane, ensuring robustness and toughness even under wet conditions. This characteristic ensures that the polymerized membrane is resistant to damage post‐implantation. The approach to constructing membranes in this manner is worthy of emulation by BP. Besides, Wang et al. designed and fabricated a double‐layer Janus nanofiber film by sequential hierarchical electrospinning [155]. Random gelatin fibers loaded with HAp are designed as internal surfaces to promote osteoblast adhesion, proliferation, and osteogenic differentiation (Figure 4B). The arranged PCL nanofibers were loaded with poly (methylacryloxyethyl trimethyl ammonium chloride‐co‐2‐aminoethyl 2‐methacrylate hydrochloride) (P(DMC‐AMA)) as the outer layer to resist the invasion of epithelial cells and bacterial infection. What is more, Gao et al. constructed a double‐layer bionic artificial periosteum by 3D printing technology, with GelMA and HAp as bionic cambium layer, and polyn‐acryloyl glycine (PACG) loaded with Mg^2+^ and GelMA's copolymer gel as bionic fiber layer [138]. Both layers contain GelMA components, which provide the basis for the realization of extrusion printing. Because HAp nanoparticles can release calcium ions, cambium can promote bone differentiation of bone marrow mesenchymal stem cells. The PACG‐GelMA‐Mg^2+^ hydrogel in the fiber layer has the mechanical strength of hydrogen bond enhancement and the degradation time of about 60 days, which plays the function of protecting the internal defect site, and the continuous release of Mg^2+^ in it shows the function of regulating the polarization of macrophages to M2 phenotype and promoting the angiogenesis of umbilical vein endothelial cells. The double‐layer artificial periosteal scaffold implanted into the critical size bone defect of the skull of rats showed the best effect of new bone formation at 12 weeks after surgery, so it had an obvious effect of promoting bone regeneration. Adhesive catechol groups can form strong bonds to tissues through hydrogen bonding, π–π interactions, and thiol reduction. In addition, the Janus periosteum may enhance bone regeneration in vivo by increasing new bone formation and new blood vessel formation.

### 5.4 Multilayer Structure

**FIGURE 5:**
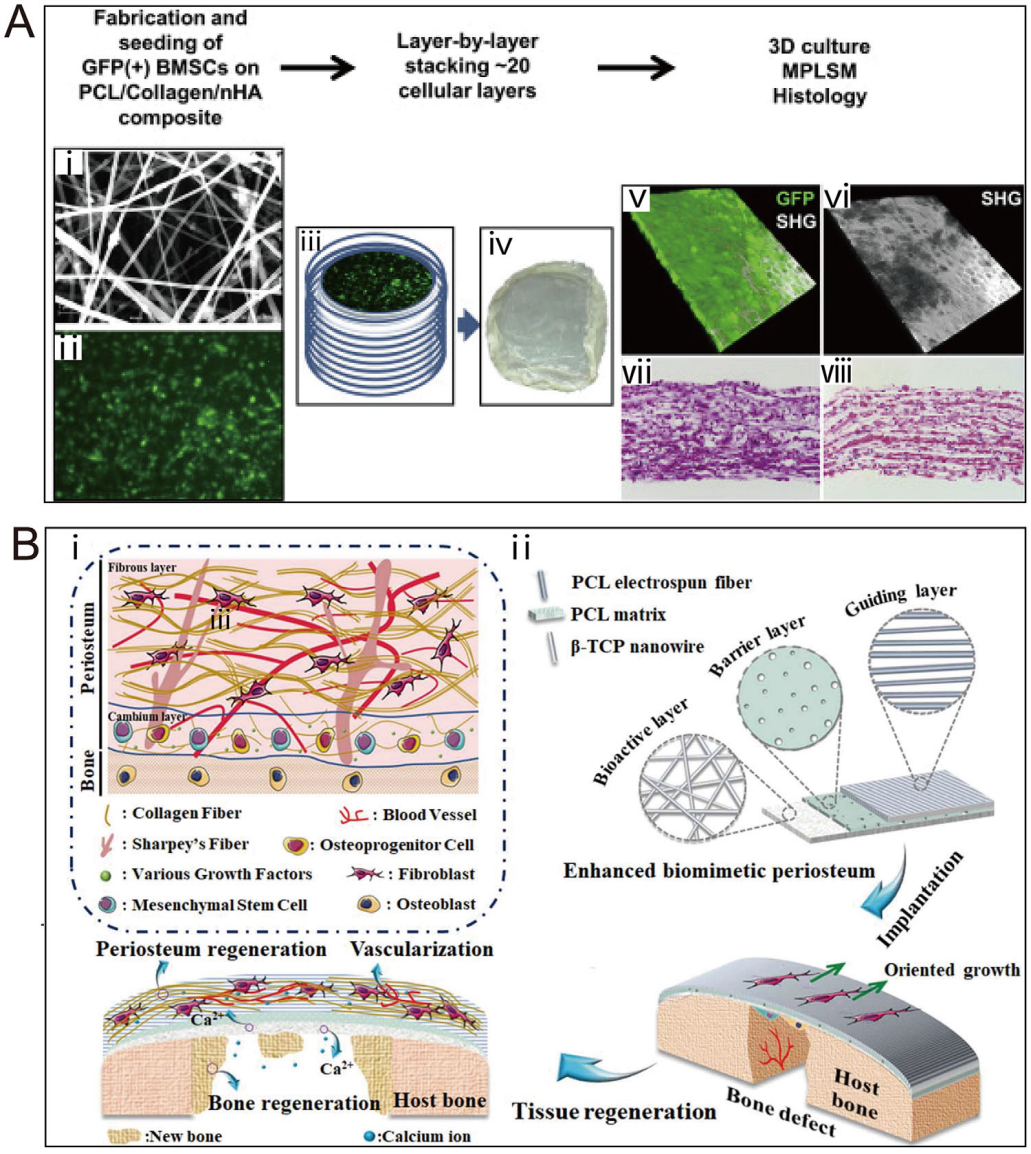
BP with multi‐layer structure. (A) Layer‐by‐layer assembly of electrostatically spun nanofiber BP structures. Layer‐by‐layer assembly of electrospun nanofibrous TEP construct. Schematic illustration of fabrication (i), seeding (ii), and assembly of the 3D nanofiber/cell construct (iii). Gross appearance of the flexible tissue membrane (iv). MPLSM images show a stacked 3D construct with GFP BMSCs (v) embedded in a rich SHG collagen matrix (vi). GFP, green, SHG, white. Histologic H&E (vii) and Picrosirius Red staining (viii) show a multi‐layered cellular construct at day 2 following assembly. Reproduced with permission [100]. Copyright 2018, Elsevier. (B) Periosteal structure (i). Structural characteristics of three‐layer reinforced BP (ii). Patterns of BP‐induced regeneration of bone and periosteal tissue (iii). Reproduced with permission [157]. Copyright 2019, IOP Publishing Ltd.

The periosteum exhibits a layered structure, and replicating the functional properties of this multilayered structure with a single structural membrane poses a challenge. To emulate the diverse functional characteristics of distinct periosteal layers and attain outcomes more closely resembling the natural periosteum, scientists have devised various multilayered BP. Multilayer nanofiber structures constructed using electrostatic spinning techniques are the most commonly used structures. Cheng et al. utilized electrospinning to prepare laminated nanofiber BP consisting of fibroin and PCL with uniform diameters. The incorporation of PCL not only improves the tensile strength of the bone membrane but also preserves the inherent hydrophilicity of SF. This multilayered structure functions as both a conduit for cell transportation and a barrier, resembling the properties of natural periosteum. Moreover, it exhibits favorable biocompatibility and osteogenic capabilities [156]. Wang et al. fabricated composite nanofiber sheets through the seeding of BMSCs on a substrate composed of PCL, collagen, and nHAp (Figure 5A). The construction of the BP proceeded layer by layer, following a bottom‐up approach [100]. The layer‐by‐layer strategy enhances the controllable integration of various functional components, imparting greater flexibility, versatility, and adaptability to the BP. Through the tailored modification of each cell layer to emulate the specific microenvironment of the periosteum, this approach establishes an effective platform for constructing BP substitutes that faithfully replicate the distinctive cellular, molecular, structural, and functional characteristics of the natural periosteum. Liu et al. engineered a three‐layer structured BP incorporating distinct functionalities [157]. Initially, they fabricated a PCL large‐pore fluffy‐oriented fiber film through electrospinning and gas foaming techniques, mimicking the fiber layer of the natural periosteum. Subsequently, β‐TCP nanowire inorganic membrane, representing the cambium of the natural periosteum, was prepared via hydrothermal synthesis and vacuum filtration (Figure 5B). Finally, the guiding layer and bioactive layer, produced by surface micro‐solubilization and recuring, were overlaid on both sides of a densely structured PCL casting film. This resulted in an enhanced BP capable of concurrently promoting in‐situ regeneration of bone and periosteum tissue. The guide layer, characterized by large pores and a fluffy‐oriented structure, interacts with soft tissue to facilitate the orientation and infiltration growth of fibroblasts, guiding the regeneration of periosteum tissue with a specific orientation. The middle PCL casting layer functions as an effective physical barrier and space maintainer, creating a stable microenvironment for the active layer at the bottom. This active layer recruits various bone cells to accelerate bone tissue regeneration. Additionally, the large porous fluffy structure of the guide layer provides a physical space for vascular regeneration facilitated by Ca^2+^ dissolved in the active layer. Tao et al. produced a microfiber BP using a water‐in‐oil PCL/carboxymethyl chitosan/sodium alginate (PCL‐CMCS/SA) emulsion prepared with Span 80 as an emulsifier. Emulsion electrospinning yielded PCL/CMCS/SA composite microfibers with an average diameter of 2.381 ± 1.068 µm, exhibiting excellent tensile strength. The composite scaffolds demonstrated low cytotoxicity, superior mechanical properties, excellent biocompatibility, and the ability to induce osteoblast bone formation [158].

## 6 BP Construction for Functional Biomimicry

This section primarily discusses some significant functional factors related to bone healing [159], summarizing the construction of BP, encompassing the inflammatory cascade reaction, biochemical factors, inorganic additives, nanodrugs, and the regulation of physiological, electrical stimulation, and innervated function (Table 3).

**TABLE 3:** Diverse artificial BP for functional biomimicry.

Classification	Construction	Experimental model	Functional biomimicry	Ref.
Inflammatory cascade	PLA/COLI/Lipo‐APY29 membrane (PCLA)	Diabetic rat skull defect	PCLA as a functional glucose‐responsive immunomodulatory adjuvant BP induces polarization of M2 macrophages and improves the inflammatory microenvironment, thus enhancing bone repair by inhibiting inflammatory signaling pathways to regulate polarization and promote the gene expression of osteogenisis and angiogenisis	[174]
	PLA/HA/Sr/Black phosphorus	Calvarial defect	The BP can fill the microcracks in the damaged bone and prevent the excessive progression of inflammation, thus promoting bone formation and angiogenesis	[43]
	PHBV/PDA‐HA/BaTiO_3_	Calvarial defect	The BP can not only promote the adhesion, proliferation and diffusion of MSCs as well as osteogenesis but also effectively induce the polarization of M2 macrophages, thereby inhibiting the ROS‐induced inflammatory response	[160]
Functional biomolecules	ICA/PCL/Gelatin nanofibers	In vitro cell validation	BP loaded with a certain amount of drug ICA can accelerate the expression of bone regeneration related proteins ALP, OCN, COL I and CA, so as to promote the good performance of bone repair	[127]
	ICA/MOX/PCL/Gelatin nanofibers	Rabbit radius defect	The rapid release of MOX in the constructed BP fiber shell can rapidly inhibit bacterial reproduction to achieve the purpose of anti‐infection. The subsequent slow and sustained release of ICA can provide a favorable femority‐related chemical signal for bone‐related cells to adhere, proliferate, and differentiate on the material	[53]
Biochemical elements				
Growth factors	BMP‐2/CS/PMA	Subcutaneous implantation	The biochemical factor BMP‐2 on BP can accelerate the induction of periosteum‐like structures, thereby replacing the natural periosteum, because of the abundance of functional periosteum‐like tissue‐derived cells, blood vessels and osteoblastic cartilage progenitors	[161]
	PLLA/PDA/VEGF/BMP‐2	Femur defect	The BP can continuously release VEGF and BMP‐2 to enhance the persistence of angiogenesis and osteogenesis during bone regeneration	[162]
Regenerative cells	MSC/osteoprogenitor cells (1:1)/PEG	/	A 1:1 mixed population of MSC and bone progenitor cells was used to better simulate the natural periosteal cell population and the production of paracrine factors to further promote allograft healing	[127]
	HUVEC/MSC cell sheets	/	The introduction of HUVECs facilitates the formation of new blood vessels, thereby vastly improving the MSC sheet properties of structure and function	[68]
Bioactive inorganic ions	Bilayer PCL/MgNH_4_PO_4_·6H_2_O (PCL/St)	Calvarial defect	The membrane's microporous layer acts as an outer layer to prevent the invasion of non‐osteogenic tissue while allowing nutrients to be transported. At the same time, the fiber layer facilitates the continuous release of bioactive struvite and the homing of mesenchymal stem cells in the lesion, thus enhancing the in situ repair of the defect	[163]
	MNBG/DA‐modified Gel/Oxidized HA (GA/HA‐BG)	Calvarial defect	BP is doped with bioactive glass that can release Ca^2+^ and SiO_4_^4−^, so it can recruit cells, promote the differentiation of MSCs into osteoblasts, and stimulate HUVECs to express endogenous vascular endothelial growth factor through PI3K/Akt/HIF‐1α signaling pathway, thus accelerating the vascularization of the defect area and synergically promoting bone defect repair	[78]
	BGN/GelMA	Calvarial defect	Different concentrations of Si^4+^ in BP can regulate the functional phenotypic transformation of Mφ, so as to stimulate the acceleration of bone repair through immune regulation	[79]
	PAA/Ce/ESM‐collagenase‐I	Subcutaneous implantation; Calvarial defect	Cerium BP can promote the early osteoclastic differentiation of macrophage cell line, promote the secretion of PDGF‐BB, VEGF and Slit3, and promote the regeneration of bone, blood vessels and nerves	[164]
Electrical stimulation	Disordered and ordered PVDF fibers (RA/AA)	In vitro cell validation	After the complete adhesion of stem cells, the self‐triggered piezoelectric effect promoted calcium ion inflow to achieve a good osteogenic effect. Stem cells on the disordered nanofibers showed better osteogenic effect due to more adhesion area and more active calcium ion signal	[165]
	CaP‐PEDOT:PSS‐MgTiO_3_‐MA (CPM@MA)	Femur defect	Nano‐conductive CPM@MA hydrogel BPs have the potential to enhance cell function by increasing endogenous TGF‐β1 and activating the TGF‐β/Smad2 signaling pathway, which can significantly promote electrically stimulated bone defect regeneration in vivo	[166]
Innervated function	PCL/Black phosphorus/DNM	Calvarial defect	Electroactive BP stimulates SCs to enter neuroprotective phenotype through Fanconi anemia pathway, enhances the AIS effect of sensory neurons, regulates DCV transport, releases neurotransmitters, and promotes the osteogenic transformation of BMSCs	[225]
	PCL/Whitlockite/Nd(III)	Calvarial defect	The continuous release of Mg^2+^ can effectively promote the upregulation of NGF and VEGF. The release of Ca^2+^ and PO_4_^3−^ ions and photothermal osteogenesis promote bone regeneration. In a combination of structure and function, the formation of nerves, blood vessels, and associated collagen greatly mimics the microenvironment of ECM and periosteum regeneration, ultimately promoting bone regeneration	[142]

### 6.1 Inflammatory Cascade

**FIGURE 6:**
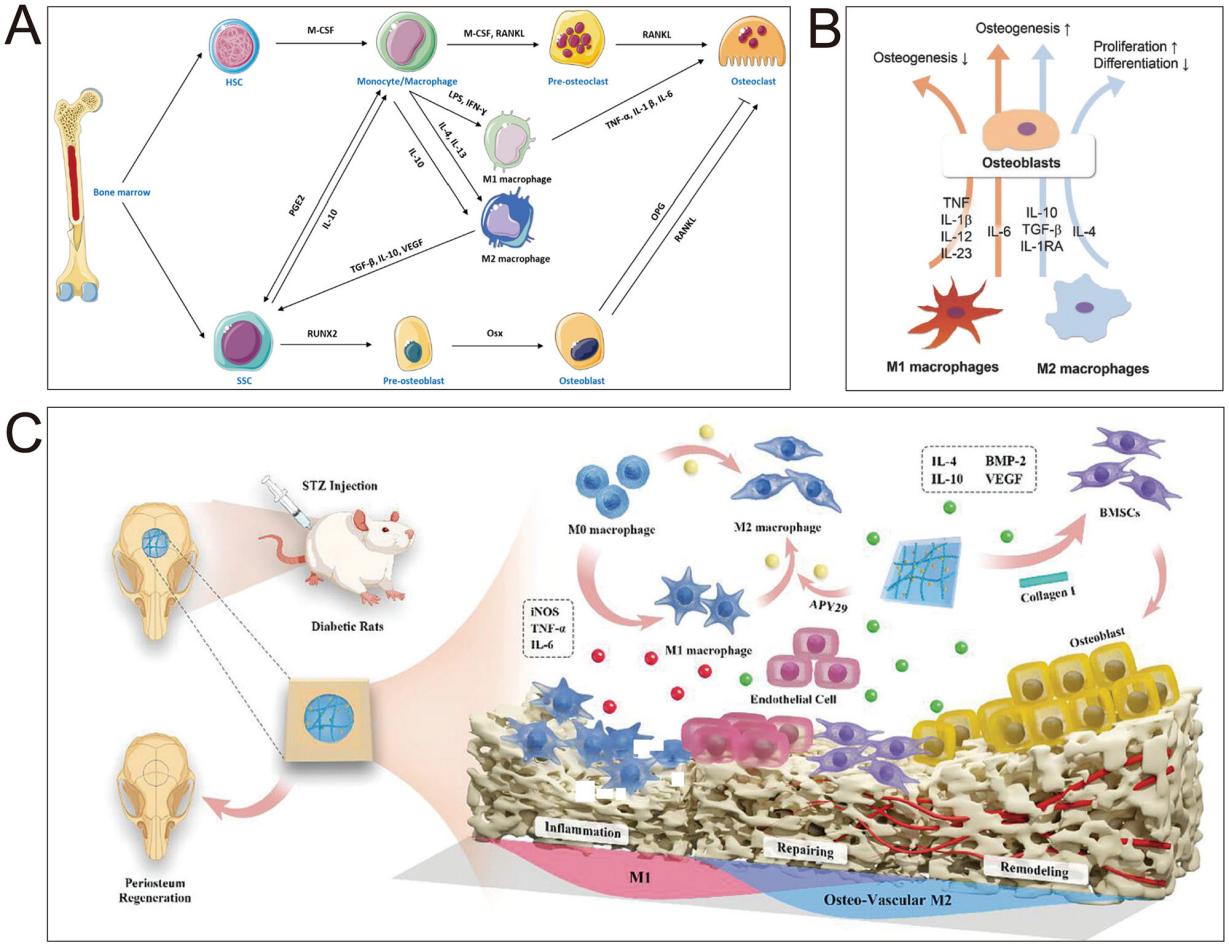
(A) Regulation of macrophage differentiation plasticity. Simplified diagram of the differentiation of a skeletal or hematopoietic stem cell into a macrophage, an osteoclast, and an osteoblast. Reproduced with permission [170]. Copyright 2022, Elsevier. (B) Illustration of macrophages' role in bone formation through the secretion of diverse cytokines. Used by courtesy of the publisher [173]. Copyright 2017, Wiley. (C) A model of a cranial bone defect in a diabetic rat, developed using PCLA nanofibers. In a glucose‐rich setting, PCLA is designed to release APY29, which adjusts the local immune milieu at the defect site. In conjunction with collagen, this fosters an increase in vascular bone reconstruction and aids in periosteal recovery. Used by courtesy of the publisher [174]. Copyright 2023, Wiley.

In the context of the periosteal microenvironment, it is crucial to address the immune regulation within the inflammatory cascade following a bone defect. The bone immune microenvironment (BIM) includes a wide range of immune cells and secreted cytokines that play a key regulatory role in bone repair [168, 169]. Bone repair of defects is a complex and well‐coordinated process through inflammation, repair and remodeling [170]. Especially the immune response of macrophages is particularly important during the inflammatory period (Figure 6A). There is sufficient evidence that immune regulation in the inflammatory phase is the key to the initial stage of bone repair. Macrophages (Mφ) act as primary mediators of the immune response (Figure 6B), with their diverse regulatory functions in bone regeneration stemming from their plasticity and heterogeneity. Traditionally, Mφ undergoes polarization into M1 macrophage subtypes under local microenvironment induction, secreting inflammatory factors such as IL‐1β, IL‐6, TNF‐α, INOS, and other cytokines, thereby promoting an early inflammatory response and osteoclast differentiation. Studies have elucidated that M1 macrophages influence bone regeneration by secreting VEGF and tumoratin‐M, which are crucial for bone healing [171]. In the intermediate and later stages of inflammation, M2 macrophages are activated, secreting molecules like arginase 1 (Arg‐1) and interleukin 10 (IL‐10) to aid tissue repair. M2 macrophages also release platelet‐derived growth factor‐BB (PDGF‐BB), matrix metalloprotein‐9 (MMP9), and BMP‐2, promoting bone formation and angiogenesis [172]. Consequently, there is a growing interest in modulating inflammation post‐bone defect by influencing macrophage polarization to enhance bone repair [173]. Qiao et al. fabricated a bionic periosteal scaffold utilizing PLA, COLI (Collagen I), and Lipo (liposome) to induce the polarization of M2 macrophages, enhancing the inflammatory microenvironment and expediting periosteal repair significantly in a model of cranial periosteal defects in diabetic rats [174]. As shown in Figure 6C, the scaffold material effectively modulates immunity in a fluctuating hyperglycemic inflammatory microenvironment, establishing a relatively stable and favorable osteogenic microenvironment. This contributes to the efficacious design of orthopaedic biomaterials for the treatment of diabetic patients. Drawing inspiration from the nanostructure and function of the periosteum, Li et al. devised a collagen membrane arranged directionally to mimic the fibrous layer of the natural periosteum to replicate the natural periosteum's fibrous architecture, which aids in the transition from the pro‐inflammatory M1 to the pro‐healing M2 macrophage phenotype [153]. In vivo experiments validated that the layered BP with immunomodulatory and osteogenic functions can serve as a substitute for autogenous periosteum grafts. Xu et al. proposed a dual‐similarity periosteum, comprising a gel phase with genepine cross‐linked carboxymethyl chitosan and collagen self‐assembled hybrid hydrogel [75]. Functioning as a barrier, this gel moderates IL‐4 dispersion during the early breach of the BP fiber phase, thus preserving a balanced pro‐inflammatory response from M1 macrophages, critical for mesenchymal stem cell attraction and angiogenesis in immediate fracture scenarios. As the gel matrix breaks down, the subsequent release of IL‐4 works in tandem with collagen to encourage a shift in macrophage polarity towards the M2 form. These macrophages then orchestrate changes in the local environment by emitting PDGF‐BB and BMP‐2, contributing to the development of blood vessels and new bone. A rat model of cranial bone deficiency confirmed the efficacy of this regulated approach to BIM management, demonstrating enhanced bone restoration efficiency through a managed sequence of inflammatory and anti‐inflammatory phases.

### 6.2 Functional Biomolecules

Once a defect forms, it becomes highly susceptible to bacterial contamination, leading to wound infection, which is detrimental to the healing process. Immediate post‐periosteal implantation sees the potential for infection and initial inflammation. Osteogenesis, however, is a prolonged process that follows inflammation. Severe early infection or inflammation can significantly impact treatment effectiveness, potentially increasing morbidity and mortality. Therefore, an optimal solution involves the controlled release of antimicrobial, anti‐inflammatory, and bone‐regenerative pharmaceuticals in a proper and orderly manner to complement the bone tissue regeneration process. Researchers load small biomolecules in BP (e.g., nanomedicines) to augment bone defect recuperation [175]. For instance, the integration of dexamethasone into BP notably advances the osteogenic differentiation of stem cells, leading to the upregulation of bone‐specific proteins and genes [176]. When combined with Icariin, the principal component of the Chinese herb Epimedium, it effectively stimulates the proliferation and differentiation of osteoblasts while concurrently inhibiting the formation of osteoclasts [53]. Alterations to the adhesive peptide RGD amplify osteoblast adhesion and dispersal across the periosteal layer, promoting bone tissue regeneration [177]. Moreover, certain drugs possessing antibacterial and anti‐inflammatory properties can be incorporated into BP. For example, Gong et al. introduced icariin (ICA) into PCL/gelatin nanofibers to prepare artificial periosteum [127]. They further introduced PEG caprolactone nucleus and gelatin shell into ICA and moxifloxacin hydrochloride (MOX), a broad‐spectrum antimicrobial drug, respectively, creating a bone and antibacterial BP through coaxial electrospinning [53]. Studies in the radius bone defect model have shown that the membrane containing dual drugs exhibits fascinating properties, offering dual antibacterial and osteogenic functions. Chu et al. successfully prepared a novel multifunctional biodegradable PLLA/gelatin‐based nanofiber membrane loaded with gallate EGCG by coaxial electrospinning, which also exhibits both antibacterial and osteogenic functions [178]. Chou et al. prepared a biodegradable PLGA nanofiber film with three embedded drugs, used as an artificial periosteum for the treatment of femoral segmental fractures [179]. This BP possesses excellent mechanical strength, concurrently enabling the prolonged release of antimicrobial drugs and ensuring tolerable bactericidal capability. The significance of this for treating infectious bone defects cannot be overstated.

### 6.3 Biochemical Elements

**FIGURE 7:**
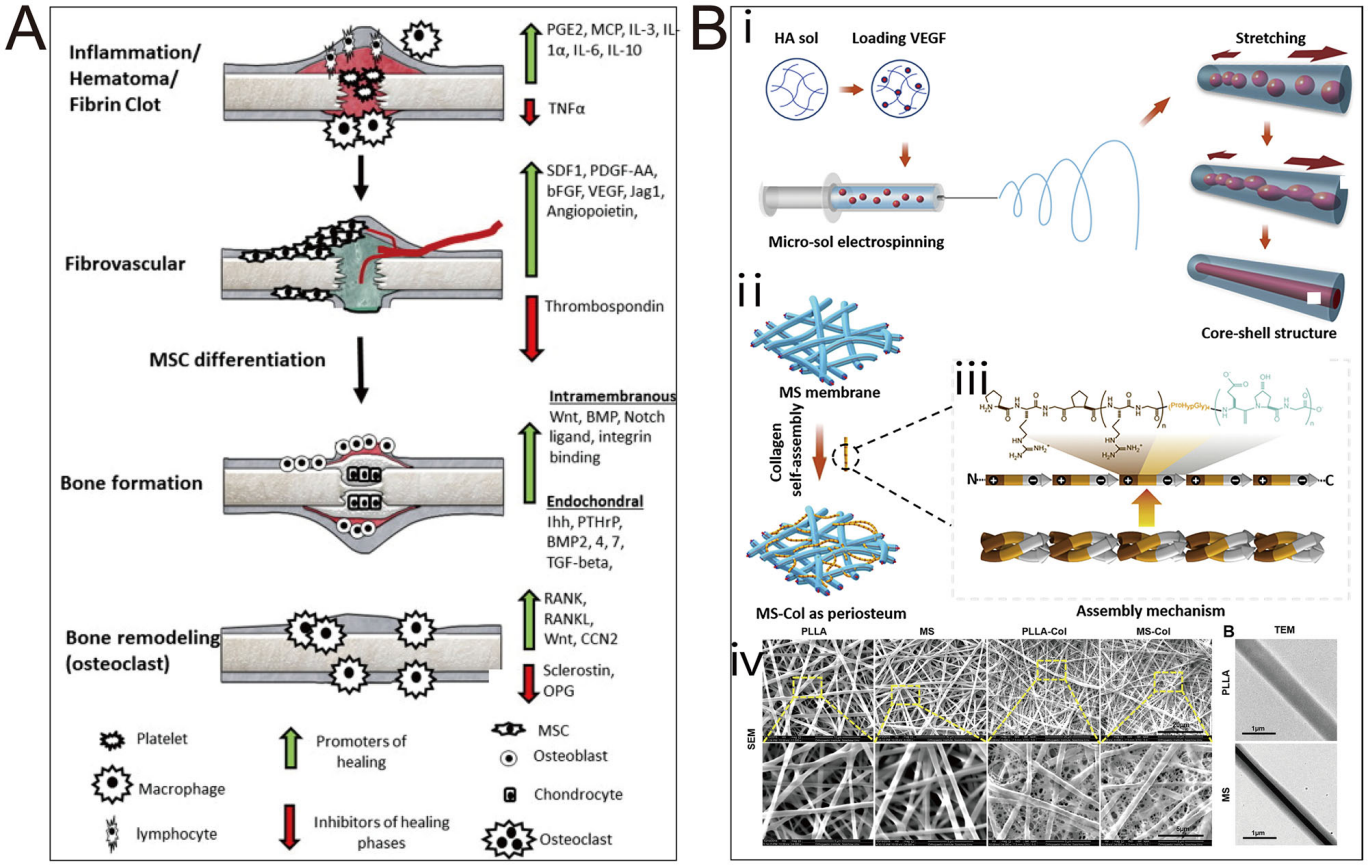
BP with functional biomolecules. (A) Phases of bone repair post‐fracture, detailing the contributory and inhibitory roles of various signaling molecules. Signaling influences are depicted with green (beneficial) and red (detrimental) arrows, illustrating their established or potential impact on bone healing. Reproduced with permission [180]. Copyright 2015, Elsevier. (B) The fabrication and structure of a stratified micro/nano‐fiber membrane. (i) Crafting VEGF‐imbued electrostatically spun membranes via HA micro‐sol electrospinning, revealing how core‐shell structures form. (ii) The multi‐tiered micro/nano‐fiber structure development process (iii) A conceptual illustration of the assembly principle and (iv) the SEM and TEM imagery of the BP, with scale bars at 20, 5, and 1 µm, accordingly. Reproduced with permission [92]. Copyright 2019, Elsevier.

Following a bone defect, the repair process involves a complex interplay of cells and growth factors, which are regulated through an array of signaling pathways as depicted in Figure 7A [180]. Growth factors such as FGF, PDGF, insulin‐like growth factor (IGF), BMP, TGF‐β, and VEGF are released from the bone lining cells, circulating platelets, and cells in the surrounding tissue [170, 181–185]. The precise activation of vascular endothelial growth factor VEGF and BMP‐2 expression is imperative to govern cellular behavior and associated signaling pathways at the defect site, thereby facilitating bone regeneration during the initial stages of healing [186, 187, 188, 189, 190]. VEGF functions to induce neovascularization, vascular sprouting, and capillary permeability, while BMP‐2 is pivotal for matrix mineralization and the promotion of new bone growth through both intramembranous and endochondral ossification processes [191, 192, 193]. The coordinated action of VEGF and BMP‐2 boosts cell growth, the development of callus, and the creation of cells essential for the endochondral ossification process [194]. Therefore, directly loading these growth factors into the BP and directly implanting them showed excellent efficacy in bone regeneration. Dai et al. employed BMP‐2 to induce the in vivo production of periosteum‐like tissue (PT), mimicking the process of endochondral ossification [161]. The PT closely mimics the natural periosteum in terms of its structure and functional characteristics. Furthermore, the addition of CS enhanced the function of PT, particularly in the recruitment of periosteum‐like tissue‐derived cells. Importantly, even when implanted into aged mice, this PT exhibited notable regenerative capabilities. Wu and colleagues utilized a dual‐technique approach, integrating micro‐sol electrospinning with collagen self‐assembly, to create a BP system with a composite layout. This design enables a controlled release of VEGF. Acting as an externally applied, vascularized fiber membrane, the BP facilitates the in vivo generation of new endogenous layers, which contribute to the restoration of both periosteum and bone tissue [90]. As shown in Figure 7B, VEGF first was added to HA hydrosol, and then the hydrosol was added to the spinning solution to form an emulsion. Through electrospinning technology, HA particles in the emulsion could be wrapped into the interior of PLLA fiber to form a core sheath structure. In the second step, the type I collagen nanofibers are assembled in the pores of the electrospun fibers to form a multi‐layer micron/nanofiber structure. VEGF has been shown in many studies to promote blood vessel formation by binding to its receptors. In this construct, the intricate hierarchical micro‐nanostructures, formed by the self‐assembled collagen fibers and PLLA fibers, effectively replicate the extracellular microenvironment. This not only safeguards the stability within and around the bone marrow cavity but also supplies both structural and biological elements crucial for cell adhesion, proliferation, and differentiation. The researchers confirmed through angiogenesis experiments that the BP containing VEGF had more vascular reticulation than the control group. Furthermore, the introduction of HUVECs has been shown to enhance neovascularization, thereby augmenting the function and therapeutic effects of tissue‐engineered membranes [80].

Currently, it has been observed that periosteal cells and MSCs secrete a substantial array of growth factors, notably VEGF and BMP‐2. As a result, directly infusing these viable cells into BP has also shown considerable therapeutic effectiveness for the repair of bone defects [195]. For instance, the direct transplantation of osteoblasts or BMSCs into the BP has been demonstrated to upregulate alkaline phosphatase (ALP) expression [71]. Additionally, MSCs can release various soluble factors, contributing to a mild inflammatory response, reduced apoptosis, and promotion of cell proliferation and differentiation. A research conducted by Hoffman et al. showcased that a BP constructed using PEG hydrogel as a platform and incorporating a mixed cell population of bone progenitor cells and BMSCs can achieve improved therapeutic effects in allogeneic bone transplantation [129]. This success is attributed to the tissue‐engineered periosteum's ability to mimic the functions of the natural periosteum and secrete numerous therapeutic factors essential for treating bone defects. Consequently, when designing such tissue‐engineered BPs, it is crucial to consider the integrated role of various types of cells in the periosteum to achieve specific periosteal functions. However, challenges such as the limited cell sources for current clinical applications, reduced cell survival post‐implantation, and potential immune responses must be addressed in future endeavors [196].

### 6.4 Bioactive Inorganic Ions

Beyond modulating the bone repair microenvironment with biochemical signals like cells and growth factors that expedite the healing process, considerable focus has been placed on innovative composite materials enriched with inorganic constituents. These composites are particularly promising as they bolster the mechanical robustness of natural polymers and amplify the biological functions of synthetic polymers [128, 129, 197]. Presently, a diverse array of inorganic additives, such as beta‐tricalcium phosphate (β‐TCP), nano‐hydroxyapatite (nHAp), bioactive glass nanoparticles (BGN), graphene oxide (GO), magnesium‐based compounds, and manganese dioxide (MnO_2_) nanoparticles, are being employed to enhance the tissue‐engineered periosteum [52, 87, 98, 151, 198, 199].

**FIGURE 8:**
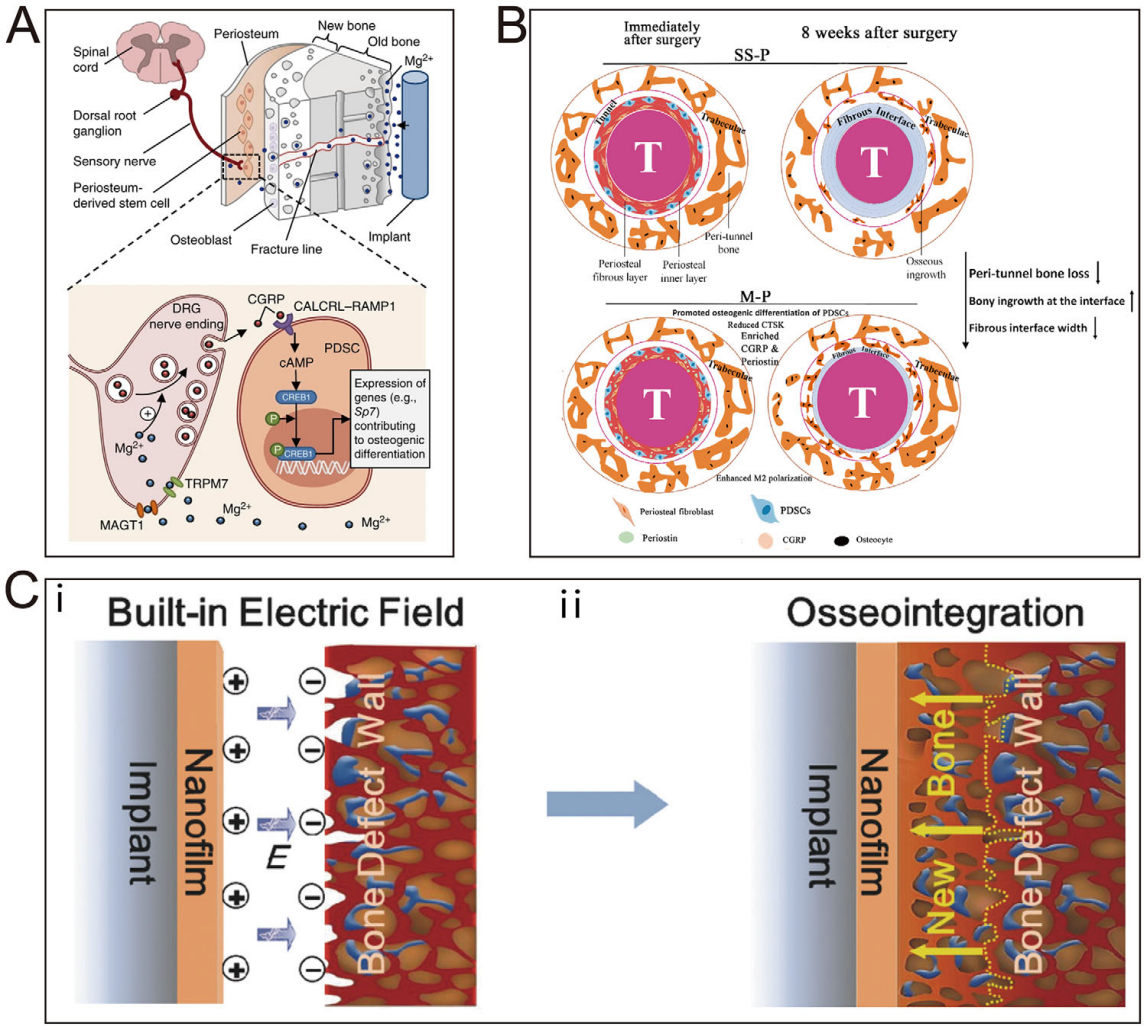
Mg^2+^ and electrical stimulation are essential for bone repair. (A) The diagram illustrates how Mg^2+^ from an implant diffuse through the bone toward the periosteum, which is permeated by sensory neurons from the DRG and populated by PDSCs that are undergoing osteogenic differentiation. In the enlarged illustration at the bottom, Mg^2+^ is taken up by DRG neurons through specific transporters, such as MAGT1 and TRPM7, causing a buildup and release of CGRP‐containing vesicles. Released CGRP then stimulates the CGRP receptor, made up of CALCRL and RAMP1, on PDSCs. This triggers CREB1 phosphorylation via the cAMP pathway, enhancing gene expression that leads to osteogenic differentiation [40]. Copyright 2016, Springer Nature. (B) Another part of the schematic details how magnesium‐pretreated periosteum aids in tendon‐bone healing. Mg^2^^+^ lead to increased expression of CGRP, periosteal proteins, and macrophage markers, promoting M2 macrophage polarization. This action diminishes bone loss at the fibrous interface and around the surgical tunnel while also fostering the formation of fibrocartilage, which is crucial for bone regeneration, particularly in the magnesium‐pretreated group compared to a control group that underwent sham surgery [42]. Copyright 2021, Elsevier. (C) The illustration also depicts how implant osseointegration is promoted through the generation of built‐in electric fields. (i) An electric field is established between the electropositive nanofilm implant surface and the electronegative wall of the native bone defect. (ii) The electric field acts as a catalyst for swift and high‐fidelity osseointegration, resulting in the implant exhibiting improved unification with the adjacent bone tissue. Reproduced with permission [214]. Copyright 2017, Wiley.

Notably, magnesium, a key element in natural bone, has garnered attention. Mg^2+^, in addition to promoting osteogenesis and osseointegration (Figure 8A), also regulates the inflammatory response [40, 42]. Recent research in bone tissue repair has underscored the role of Mg^2+^ in facilitating neurogenesis. These studies have revealed that Mg^2+^ can stimulate the production of CGRP‐α in sensory nerve neurons, with CGRP aiding endothelial cell movement and angiogenesis in a dose‐dependent way [200]. Concurrently, CGRP activates focal adhesion kinase (FAK) at the Y397 site and amplifies VEGF‐A activity. This action leads to enhanced osteogenic differentiation in stem cells and encourages the migration of endothelial cells (Figure 8B). Therefore, another study has treated periosteum with magnesium, demonstrating that such treated periosteum can boost the expression of CGRP in neurons following implantation, thereby fostering increased osteogenic differentiation of periosteal stem cells and aiding in the bone healing process [59]. Particularly noteworthy is the bidirectional interaction with piezoelectric or electrical materials, which can further enhance the neural differentiation of BMSCs. Wang et al. demonstrated that, under the combined influence of piezoelectricity and sustained release of Mg^2+^, BMSCs exhibited strong synergistic effects in terms of neurogenesis, angiogenesis, and osteogenic differentiation [201]. This opens up a new avenue for the future construction of bone substitute materials with innervation capabilities. Thus, some studies have shown that whitlockite nanoparticles (WH NPs: Ca_18_Mg_2_(HPO_4_)_2_(PO_4_)_12_) can reproduce early bone regeneration by increasing Ca^2+^ and PO_4_^3−^ ion concentrations and inhibiting osteoclast differentiation [202, 203]. At the same time, the sustained release of Mg^2+^ in WH has been shown to promote the secretion of NGF and VEGF [204, 205]. Therefore, it is not difficult to find that the introduction of bio‐functional inorganic ions can promote neurogenesis, angiogenesis and bone formation. Qin et al. developed an innovative approach for bone defect repair by constructing a periosteal bandage for transplantation [206]. They incorporated MgO NPs into the bandage through electrostatic spinning, using PCL as a substrate. Subsequently, a BP with a micro‐nano layered structure was successfully created by allowing collagen to self‐assemble on the bandage's surface. The study demonstrated that the release of Mg^2+^ played a crucial role in promoting the repair of bone defects. This was achieved by modulating immune cells, facilitating blood vessel formation, and promoting nerve growth. Overall, Qin et al.’s work introduces a novel, intracellularly activated strategy for effectively addressing bone defects.

Besides, HAp is structurally similar to the mineral components of natural bone and has found extensive applications in bone restoration and dental implants. Typically, HAp is frequently employed as a composite filler to enhance the mechanical properties and bioactivity of polymers [207]. For instance, incorporating HAp into gelatin substantially enhances the mechanical properties of the composite and retards the degradation of gelatin. Moreover, the released Ca^2+^ ions can expedite the mineralization process, thereby augmenting osteogenesis [208]. Similarly, BGN is commonly incorporated into BP as nanofillers to enhance their overall properties. Incorporation of BGN into synthetic polyvinylidene‐trifluoroethylene (PVFT) polymers significantly enhances bone cell attachment and proliferation and promotes endothelial cell angiogenesis, thereby accelerating bone tissue reconstruction [209]. In addition, conductive GO induces osteogenesis in stem cells and facilitates the enhancement of neurogenesis in the process of bone repair [52, 98]. The incorporation of MnO_2_ into BP can create a favorable microenvironment for bone remodeling. This is achieved by leveraging the anti‐inflammatory effects of manganese ions to alleviate early inflammation [198]. Magnesium phosphate minerals (such as guanite, calcium magnesite, and amorphous products) have been proposed as promising alternatives to classical calcium phosphate bone substitutes due to their more suitable degradation rates and intrinsic osteoconduction and osteoinduction [163]. Zhang et al. constructed BP with low, medium and high content of inorganic ions using organic–inorganic double crosslinking techniques of BGN and GelMA with different content [79]. The release concentration of Si^4+^ in BP was adjusted by changing the content of BGN in BP to study the ability and mechanism of BP to regulate immune repair. When BGN concentration was 3 wt% (MM@G), the artificial periosteum showed programmed M2 polarization, which showed a certain ability of bone immune regulation. Under the premise of inhibiting the phosphorylation of inflammatory pathway signaling proteins, artificial periosteum has both BMSC recruitment and angiogenesis functions, and there is a synergistic relationship between bone immune microenvironment and bone repair. It is suggested that appropriate concentration of inorganic ions is important to promote bone repair. Yang et al. used dopamine‐modified gelatin and oxidized hyaluronic acid (OHA) to prepare BP with bone tissue self‐adhesive function and similar to extracellular matrix, and further mixed micro/nano bioactive glass into hydrogel to prepare organic/inorganic co‐crosslinked hydrogel film (GA/OHA‐BG) [78]. GA/OHA‐BG composite hydrogel periosteum has good adhesion to bone tissue and forms a stable barrier in the bone defect area. In addition, the release of Ca^2+^ and SiO_4_^4−^ ions accelerates angiogenesis in the defect area and creates a microenvironment conducive to the adhesion, proliferation and osteogenic differentiation of mesenchymal stem cells, as well as the further reconstruction of new bone, thereby inducing the healing of critical size bone defects.

Deferoxamine (DFO) is an iron chelating agent that has been widely used as an anoxic simulation compound under normoxic conditions [134]. It has been reported that when DFO is used in bone defect models, it can accelerate angiogenesis and bone regeneration by regulating the expression of hypoxia inducible factor‐1 α (HIF‐1α) to stimulate angiogenesis and osteogenesis related genes [210, 211, 212]. He et al. demonstrated a strengthened anisotropic complex hydrogel consisting of chitin chains and maleated chitin whiskers (mCHW) through a dual chemical and physical cross‐linking strategy and a stretching‐drying process, followed by the selection of bioactive DFO for further functionalization to enhance its mechanical viscoelasticity and osteogenic and vascularizing capabilities [213]. This design not only allows the composite hydrogel to have a significant tensile strength (17.6 ± 1.4 MPa) and Young's modulus (28.1 ± 2.2 MPa). Moreover, it can effectively enhance cell adhesion and proliferation, and has excellent ability to promote cell osteogenesis and angiogenesis. In addition, in order to simulate the role of natural periosteal microbe in bone repair, Wan et al. used the principle of biomimetic mineralization to construct a cerium (Ce)‐containing BP by embedding cerium oxide in the eggshell membrane (ESM). Ce‐based BP can promote the early osteoclastic differentiation of macrophage lineage cells and the secretion of PDGF‐BB, VEGF and Slit3, and promote the regeneration of bone, blood vessels and nerves [164].

### 6.5 Electrical Stimulation

The bioelectric properties of the periosteal microenvironment are irritant to cell proliferation, adhesion and differentiation. Therefore, it is impressive enough to design and manufacture BP that adapts to and interacts with the internal environment, inspired by some special cues from the internal environment of the organism. The endogenous electrical stimulation of the bone microenvironment is revealed by piezoelectric properties. The endogenous electric field of bone was discovered early on, and the intrinsic piezoelectric attributes of bone contribute significantly to the feedback regulation of both bone regeneration and remodeling. Moreover, electric fields have been shown to direct cell movement towards electrodes and modulate cellular proliferation, locomotion, and adherence, indicating that cells are responsive to and can be propelled by electric fields. Electric fields are known to steer cellular migration towards electrodes and influence cellular growth, motility, and bonding, indicative of cells' responsiveness to and ability to be mobilized by electric dynamics [215, 216]. Hence, employing precise electric fields could positively impact cellular behavior. For instance, electrical communication within the body can orchestrate macrophage dynamics such as migration, phagocytosis, and cytokine release [217]. Furthermore, recent studies have identified a variety of physiological factors—mechanical stress, electrical forces, and magnetism‐as promising elements for advancing bone regeneration, by shaping the activities and developmental stages of bone‐associated cells [218, 219]. For example, bone tissue itself, due to its piezoelectric characteristics, is inherently energized by the body's mechanical actions (Figure 8C), which can then regulate the metabolic processes and proliferation of bone cells.

Calcium concentrations serve as a key conduit for the electrical stimulation of neuronal cells. Such stimulation has been observed to escalate the activity within calcium‐dependent pathways like MAPK and cAMP, leading to synchronized membrane activity, the creation of growth cones, and the initiation of neural repair [220]. Furthermore, this stimulation governs the structural framework of neurons by adjusting microtubules, microfilaments, and expediting the transport of axonal proteins, all of which are essential for the growth of axons and ultimately for fostering nerve regeneration. In the context of bone cells, electrical stimulation has evidenced favorable outcomes on the cellular dynamics of osteoblasts, which encompasses various cell types such as BMSCs, BPCs, osteoblasts, and endothelial cells, promoting their expansion, migration, and evolution [221]. The promotion of bone generation by electrical stimulation is potentially attributed to enhanced intracellular calcium levels within osteoblasts, activation of voltage‐gated calcium channels, and a surge in osteogenic activity driven by the calmodulin signaling pathway. [222] Hence, addressing bone deficiencies effectively necessitates the repair of the periosteum, teeming with nerves, to restore the intrinsic electric field critical for such regenerative processes.

Notably, during the proliferation and adhesion of BMSCs to the fibers, mature focal adhesion (FA) is not fully formed, preventing cells and the substrate from perceiving each other's mechanical behavior. As a result, cell activity causes cells to slide relative to the substrate without deforming the nanofibers. After the formation of mature FA, biophysical signals within the cells are conveyed outside through integrin‐mediated force conduction, leading to the deformation of the fiber network. This, in turn, generates a piezoelectric potential as a feedback signal due to cell traction, promoting the differentiation of stem cells [223]. When BMSCs were cultured on ordered PVDF nanofibers under this in situ electrical stimulation for 7 days, they successfully differentiated into neuron‐like cells without the need for external mechanical or electrical stimulation [116]. This emphasizes the considerable potential of piezoelectric fibers in promoting the differentiation of stem cells into both bone and nerve cells. Additionally, the advancement of intelligent bone plating systems may benefit from incorporating on‐site, as‐needed electrical stimulation driven by the piezoelectric potential resulting from cellular traction. This approach represents a promising direction for future innovations in bone repair technologies.

### 6.6 Innervated Function

**FIGURE 9:**
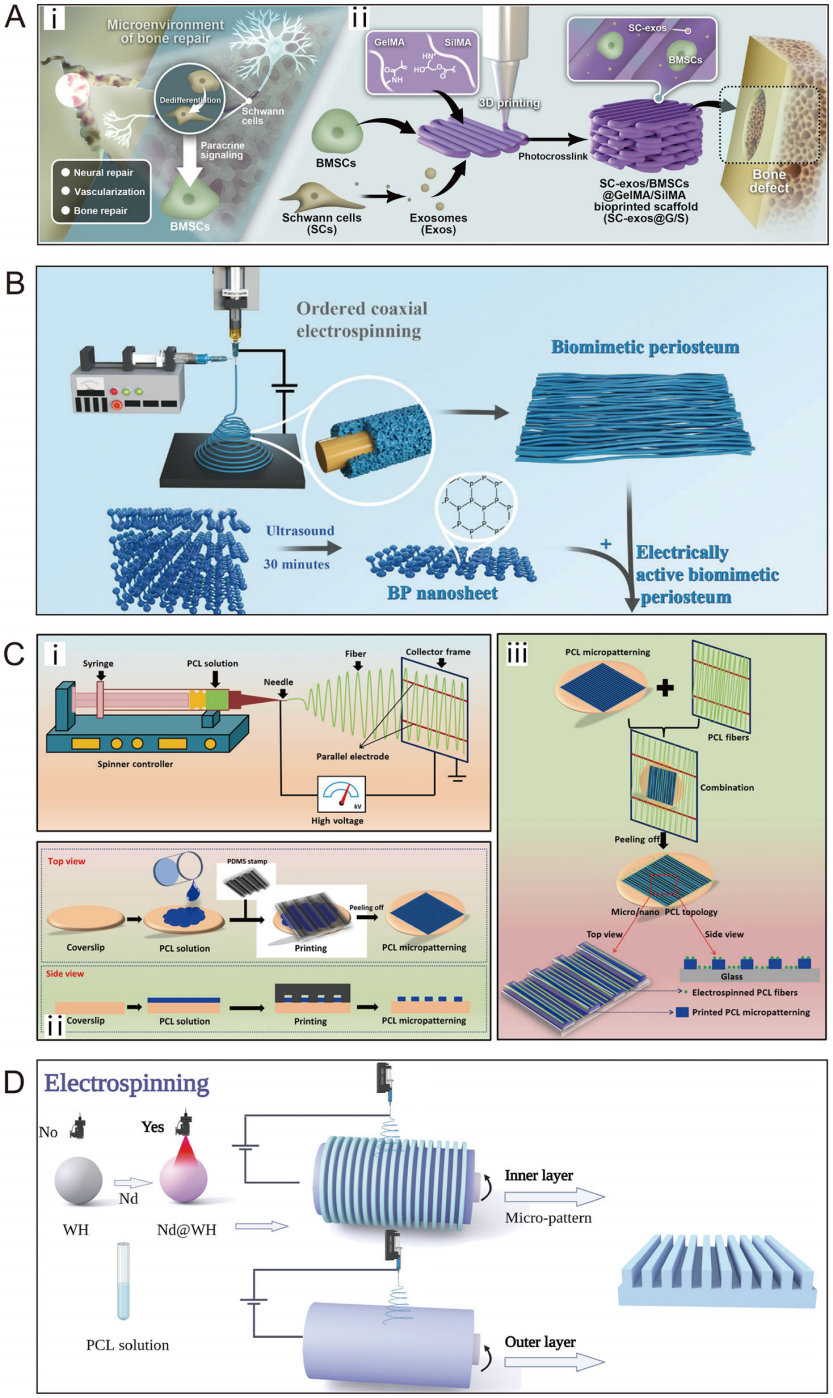
Composites with neurogenesis. (A) Assessment of β III‐tubulin and S100β expression in rat bone tissue before and after injury. (i) HE staining of bone tissue before injury. (ii–iv) Immunofluorescence staining for β III‐tubulin (red) and S100β (green) with DAPI (blue) at 3, 7, and 14 days post‐fracture, respectively. Scale bars: 1 mm (i1,i2); 200 µm (i3) [224]. Copyright 2023, Elsevier. (B) Schematic diagram illustrating the preparation and study of electroactive periosteum for the repair of bone defects after implantation. Reproduced with permission [225]. Copyright 2023, Wiley. (C) The schematic diagram illustrates the preparation process for anisotropic micro‐ and nanocomposite topologies. (i) Electrostatically spun PCL oriented fibers. (ii) Manufacturing of PCL micropatterns. (iii) Combination of PCL oriented fibers and PCL micropatterns to form a micro‐and nanocomposite topology. Reproduced with permission [226]. Copyright 2021, American Association for the Advancement of Science. (D) Construction of photothermal bilayer bionic periosteum and schematic diagram of in vivo treatment in SD rats. Reproduced with permission [167]. Copyright 2021, Elsevier.

In our discussion of BP construction strategies, we have emphasized the importance of replicating the structural and functional characteristics of the natural periosteum, including its angiogenic and osteogenic capabilities. The role of nerves in the periosteum as the primary coordinator and participant in immune responses and angiogenic bone regeneration at the site of bone injury should not be underestimated. They release neurotrophic factors that can influence the behavior of nearby cells and promote the overall healing process [227, 228]. However, research dedicated to enhancing the innervation capacity of the periosteum is not comprehensive, yet the innervation capacity of the periosteum is crucial for the comprehensive regeneration of bone. In existing studies, Schwann cells (SCs) have been identified as the seed cells in the peripheral nervous system responsible for innervating nerves crucial to bone regeneration [227, 229, 230]. The deficiency of SCs has been directly linked to delayed bone tissue regeneration. In the absence of injury to the skeletal system, only a minimal amount of SCs is present at the periosteal sites in the bones (Figure 9A). However, after the occurrence of a bone defect, a significant secretion of SCs takes place at the defect site over time [228]. This suggests that SCs are directly involved in the healing process of bone defects at the site of injury. Su et al. initially synthesized targeted phosphatidylserine (PS) exosomes by employing the self‐assembly effect to bind PS with exosomes from SCs [231]. Subsequently, a BP with neurogenesis, angiogenesis, and bone regeneration properties was fabricated by loading exosomes and polyethyleneimine (PEI) onto the electrostatically spun fibers of PCL using the electrostatic effect. In a weakly acidic environment (pH = 6.7), exosomes can be abundantly released to directly target the site of nerve axon injury, thereby promoting axonal regeneration. Additionally, this BP not only stimulates angiogenesis but also enhances the expression of several osteogenic genes. In vivo experiments further substantiate this claim. The strategy of utilizing SCs exosomes in conjunction with other materials to construct bionic BPs with innervation ability holds significant value. In another study, they proposed a new strategy for constructing innervated BPs [225]. They loaded two‐dimensional black phosphorus nanosheets onto PCL fibers with oriented structures by electrostatic forces. The electroactivity of the black phosphorus nanosheets coupled the BP to the bone (a natural endogenous electric field) could stimulate SCs to enter the neuroprotective phenotype through the Fanconi anemia pathway, enhance the axon initial segment (AIS) effect of sensory neurons, regulate the dense core vesicles (DCV) transport, release neurotransmitters, and promote bone formation transformation of bone marrow MSCs, thus confirming that this electroactive periosteum could promote bone regeneration through sensory nerves (Figure 9B). This is one of the few electrically active BPs currently available, and their study opens the door for the development of other electrically active BPs.

Surface morphology is also a crucial method for regulating nerve regeneration. It has been reported that grooves with an oriented topology can influence the alignment orientation during the proliferation of SCs and consequently promote SC proliferation [232]. Furthermore, the width of the grooves had a more substantial impact on SCs compared to the height of the grooves. In a study conducted by the Shi team, it was demonstrated that the chitosan grooves in the 30/30 spine structure could more effectively guide the alignment of SCs without affecting the physiological function of SCs [233]. Subsequently, they integrated electrostatic spinning and recess molding techniques to fabricate neural implants featuring micro‐ and nanostructures. While their exploration was limited to applications in the peripheral nerves field, the implant structurally resembled the gradient structure of periosteum in their study [226, 234]. This micro‐nanometer hierarchy is worth learning from in our future research on building BPs (Figure 9C). Unfortunately, a singular topology lacks the capability to effectively promote the biological functions of SCs, particularly in terms of the secretion of NGF [235]. NGF is instrumental in steering the development of sensory nerves and significantly contributes to the process of bone renewal by specifically attaching to the neurotrophic tyrosine kinase receptor type 1 (Trk‐A) [236]. Throughout the bone defect healing process, NGF exhibits a continuous elevation until reaching its peak, underscoring its pivotal role in the entire bone repair process [237]. Moreover, NGF is intricately linked to bone‐forming cells and the vascular system [238]. Consequently, NGF promotes bone healing by directly stimulating osteoblasts and indirectly recruiting sensory nerves that transport osteogenic factors. Therefore, exploring methods to stimulate SCs to produce more NGF may prove to be a valuable avenue for investigation in the construction of BPs. Li et al. integrated micro and nano morphologies, bioactive ions, and photothermal effects to construct BP with combined angiogenesis‐neurogenesis and bone regeneration [167]. Its inner layer is nanofibers with oriented groove structure, which ensures the proliferation of osteoblast adhesion. The outer layer is a conventional electrostatically spun fiber, which avoids the growth of soft tissues towards the bone defect site. And then the osteogenic function of BP was ensured by introducing WH NPs (Figure 9D). The doping of Nd(III) in WH as an inducer of photothermal reaction further enhanced its osteogenic function under photothermal conditions. Meanwhile, accompanied by the continuous release of Mg^2+^ in WH, it could effectively promote the neuronal cells to produce more NGF. Future research should concentrate on developing BPs that replicate not only the physical and biochemical characteristics of the natural periosteum but also its neural components. This will necessitate the incorporation of neural guidance channels, materials that release neurotrophic factors, and potentially neural progenitor cells into BP constructs. These innovations aim to enhance the innervation capacity of BPs, thereby offering a more complete and effective solution for bone repair and regeneration.

## 7 Conclusion and Future Perspectives

This article delves into the intricate realm of BP, a frontier in bone defect repair and regenerative tissue engineering. The natural periosteum, with its pivotal role in bone dynamics, serves as a blueprint for the design of BP. The review meticulously examines the structure and function of the periosteum, laying the groundwork for understanding the complexity of its biomimetic counterpart. The incorporation of innovative materials, structural designs, and functional strategies has shown great promise in mimicking the complex architecture and biological functions of natural periosteum. However, addressing the outlined limitations and challenges is crucial for the translation of these advances from the laboratory to clinical practice, several persisting limitations and challenges warrant further investigation to enhance efficacy and applicability:

1. Customizability: Although current biomaterials exhibit good biocompatibility and bioactivity, variations in specific factors such as patient age, health status, and chronic diseases can lead to differences in tissue response. Therefore, a one‐size‐fits‐all approach may not be suitable for BP. Developing customizable BP approaches that are tailored to meet individual patient needs is a challenge that requires further research.
2. Neurovascular integration and mimicking the complexity of natural periosteum: A critical aspect of the success of BP is the development of integrated neurovascular networks that ensure sufficient nutrient supply, waste removal, and overall tissue integration and healing. The natural periosteum is a complex structure with a unique combination of biochemical, biomechanical, and cellular components. Current strategies often fall short in replicating both the intricate neurovascular networks and the overall complexity found in natural periosteum, limiting the thickness, viability, and functionality of engineered tissues. Future research should focus on developing multi‐component systems that not only facilitate the formation of neurovascular networks but also mimic the natural periosteum's complexity. Innovations in vascularization techniques, incorporation of angiogenic factors, and strategies for nerve growth and integration are vital. By closely mirroring the natural healing processes and the multifaceted nature of periosteum, these advanced biomimetic constructs could dramatically enhance the regenerative capabilities of biomaterials used in bone repair and regeneration.
3. Immune response and inflammation: The interaction between the implanted BP and the host immune system can significantly influence the healing process. Uncontrolled inflammatory responses may lead to fibrosis, implant rejection, or failure of bone regeneration. Developing strategies to modulate the immune response and promote a conducive environment for bone healing is a vital area of research.
4. Degradation rate: The degradation rate of the BP should be synchronized with the rate of new bone formation to ensure that the scaffold provides support throughout the healing process. Achieving this synchronization is complex and requires a deep understanding of the degradation kinetics of the materials used.
5. Cost and scalability: The production of BP involves sophisticated materials and techniques, which can be costly. Making these technologies affordable and scalable for widespread clinical use remains a challenge.

Future research should focus on the development of multifunctional biomaterials that offer enhanced mechanical properties, biocompatibility, and bioactivity tailored to individual patient needs. Advances in 3D bioprinting, nanotechnology, and tissue engineering hold the potential to overcome current obstacles in vascularization and integration. Moreover, interdisciplinary collaborations among material scientists, biologists, engineers, and clinicians are essential to refine these biomimetic approaches and realize their full potential in regenerative medicine. As we continue to explore the intricacies of bone healing and regeneration, the pursuit of an ideal BP remains a dynamic and evolving field. The lessons learned from current challenges will undoubtedly pave the way for innovative solutions that bring us closer to achieving successful bone repair and regeneration for patients worldwide.

## Conflicts of Interest

The authors declare no conflict of interest.
